# Recent Developments in the Immobilization of Laccase on Carbonaceous Supports for Environmental Applications - A Critical Review

**DOI:** 10.3389/fbioe.2021.778239

**Published:** 2021-12-06

**Authors:** Younes Adamian, Linson Lonappan, Komla Alokpa, Spiros N. Agathos, Hubert Cabana

**Affiliations:** ^1^ Université de Sherbrooke Water Research Group, Department of Civil and Building Engineering, Université de Sherbrooke, Sherbrooke, QC, Canada; ^2^ Laboratory of Bioengineering, Earth and Life Institute, Catholic University of Louvain, Louvain-la-Neuve, Belgium

**Keywords:** immobilization, biochar, micropolluants, environmental contaminants, laccase

## Abstract

Τhe ligninolytic enzyme laccase has proved its potential for environmental applications. However, there is no documented industrial application of free laccase due to low stability, poor reusability, and high costs. Immobilization has been considered as a powerful technique to enhance laccase’s industrial potential. In this technology, appropriate support selection for laccase immobilization is a crucial step since the support could broadly affect the properties of the resulting catalyst system. Through the last decades, a large variety of inorganic, organic, and composite materials have been used in laccase immobilization. Among them, carbon-based materials have been explored as a support candidate for immobilization, due to their properties such as high porosity, high surface area, the existence of functional groups, and their highly aromatic structure. Carbon-based materials have also been used in culture media as supports, sources of nutrients, and inducers, for laccase production. This study aims to review the recent trends in laccase production, immobilization techniques, and essential support properties for enzyme immobilization. More specifically, this review analyzes and presents the significant benefits of carbon-based materials for their key role in laccase production and immobilization.

## Introduction

Water is one of the fundamental resources on which all life on earth is anchored. Over the past few decades, concerns regarding the shortage in freshwater supply and its effect on the sustainability of human societies have increased (Rathi et al., 2021). Rapid population growth, industrialization, climate change, and environmental destruction are factors directly involved in increasing water demand (Jéquier and Constant, 2010; Rathi et al., 2021). Water recycling and reuse through proper treatment is a potential solution to meet the current and rising water demand. In this process, polluted water from different sources including households, industries, hospitals and agriculture may be treated to an acceptable standard and recovered for further use (Englande et al., 2015). However, non-regulated micropollutants termed emerging contaminants (ECs) such as pharmaceuticals and personal care products, certain pesticides, food additives and synthetic hormones constitute a major challenge to existing water treatment methods (Taheran et al., 2018).

ECs is a standard term created to identify environmental risks of pollutants released into the environment with unpredictable consequences (Rathi et al., 2021). According to the United Nations Educational, Scientific and Cultural Organization (UNESCO), the term ECs refers to a group of natural or synthetic chemicals or microorganisms with known or suspected negative effect on humans’ health or the environment (UNESCO, 2019). The word “emerging” does not imply the pollutants that are recently accumulated in the environment; in contrast, this term defines the concern and awareness regarding their negative impacts that are emerging in the world (Scaria et al., 2021). The best-known and widely occurring ECs are hormones such as contraceptives, personal care products such as fragrances and deodorants, pesticides such as insect repellents, and pharmaceutical compounds such as painkillers. At hospital wastewaters, landfills, municipal sewage, fertilizer industries, pharmaceutical production plants, concentrations of ECs could be detected (Ahmed et al., 2017). Up to now, there is no regulation regarding ECs concentration in the environment but several attempts can be found in Europe and North America to reduce their released levels (Taheran et al., 2018). For instance, in Canada and Switzerland, different projects have proposed potential strategies to reduce EC concentration in wastewater treatment plants (WWTPs) (Morales-Caselles et al., 2016; Schmidt, 2018).

**FIGURE 1:**
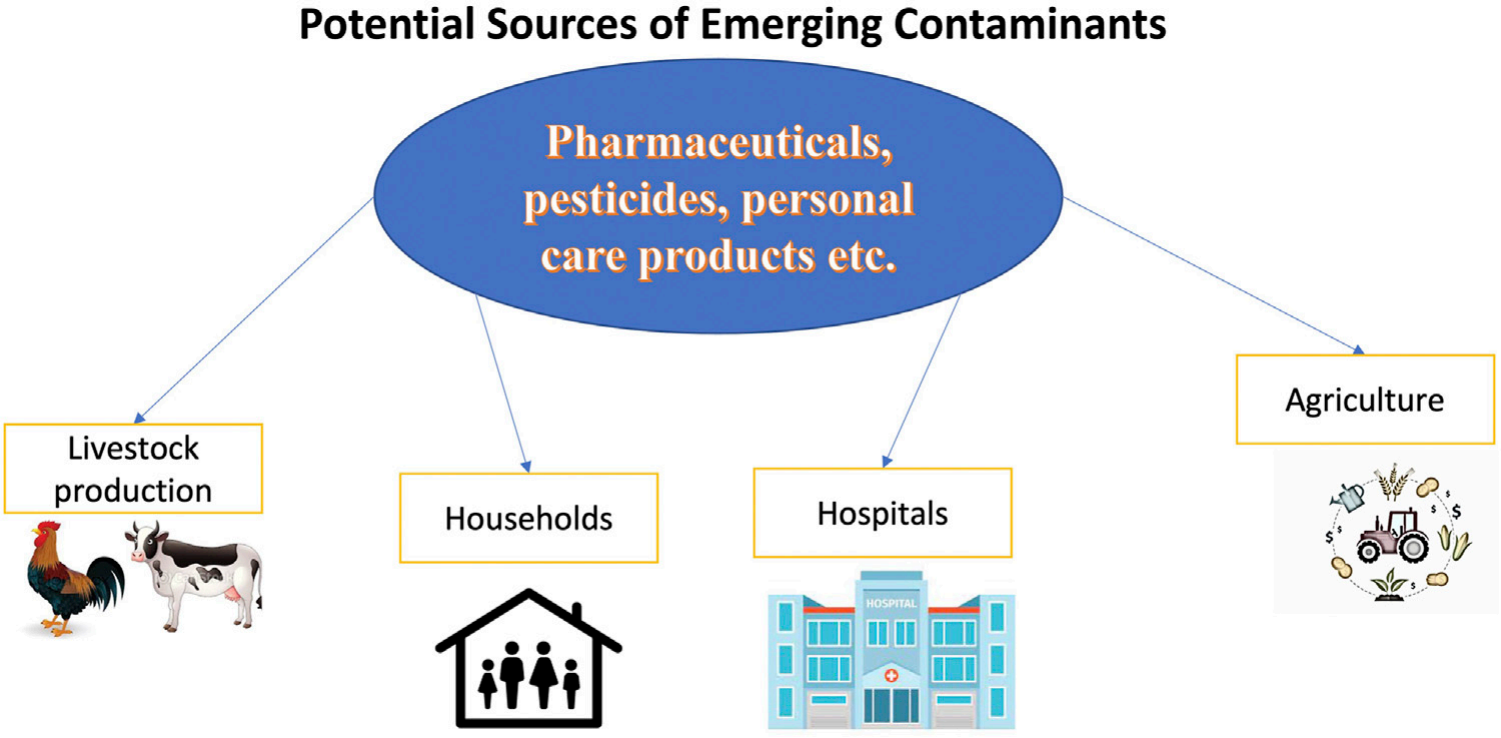
Routes for EC spreading in the environment.

Usually, EC concentrations in the environment range from parts per trillion (ppt or ng L^−1^) to parts per billion (ppb or µg L^−1^) (Petrie et al., 2015; Rout et al., 2021). Figure 1 demonstrates routes of EC spread into the environment (Gomes et al., 2020).

Conventional WWTPs are not capable of properly removing all ECs especially pesticides, detergents, pharmaceuticals and personal care products (PPCPs) at ng L^−1^ or µg L^−1^ from the wastewater and, consequently, ECs will get discharged into the environment (Mohapatra and Kirpalani, 2019). These pollutants could last for a long period of time and circulate, migrate, and transform in the different environmental matrices (Tran et al., 2019). Previous studies have demonstrated that the ECs might be found in conventionally treated wastewater, urban sewage, agricultural runoff, freshwater, and drinking water (Husk et al., 2019; Tran et al., 2019).

The existence of ECs in the environment is a global concern since in the long run their presence could have adverse effects on living organisms (Gomes et al., 2020). These could include bacterial resistance, feminization of aquatic organisms, neurotoxicity, endocrine disruption, and cancer along with other unidentified adverse effects (Mohapatra and Kirpalani, 2019). Several studies have explained the possibility of animal behavior alteration due to exposure to ECs. For instance, Barry (2014) found that tadpoles (*Bufo arabicus*) became more vulnerable to predation after exposure to fluoxetine (concentration around 3 µg L^−1^). In Denmark, from 1993 to 2006 a study demonstrated that exposure of patients 56–61 years old to Perfluorooctanoic acid (PFOA) and Perfluorooctane sulfonate (PFOS) could lead to cancer development (Lei et al., 2015).

Even though the concentration of ECs in the environment is relatively low, they still could affect negatively the food chain. Consequently, it is important to understand how to eliminate them from water and wastewater. EC removal methods may be categorized into four different groups, namely physical (such as sedimentation, precipitation, adsorption, and filtration), chemical (such as ozonation, photolysis, and Fenton), biological (such as activated sludge, aerobic microbial treatment, and enzymatic treatment), and hybrid systems (Ahmed et al., 2017; Taheran et al., 2018). Table 1 summarizes the limitations and advantages of each procedure. Among these four categories, biological treatment can be identified as an eco-friendly and cost-effective methodology. In this approach, large molecules could be degraded into smaller ones using different microorganisms such as bacteria, fungi, and algae (Unuofin et al., 2019).

**TABLE 1:** Advantages and challenges of treatment procedures for ECs removal.

Treatment process	Advantages		Limitations	Reference
Physical process				
Adsorption		Wide range of available adsorbents for different pollutants	Generate secondary pollution (solid waste)	Varsha et al. (2022)
			The existence of organic matter can affect the performance negatively	(Sophia and Lima, 2018)
Reverse osmosis		High removal efficiency for PPCP and EDC removal	High operation and maintenance cost	Egea-Corbacho Lopera et al. (2019)
				Rai and Shrivastav, (2022)
Biological Treatment Process				
Activated Sludge		Environmentally friendly	Not applicable for wastewaters with COD >4000 mg L^−1^	Koumaki et al. (2021)
		Low operational and maintenance cost		
Microbial reactor		High removal efficiency	Low removal efficiency for pharmaceutical compounds	Mery-Araya et al. (2019)
		Environmentally friendly		Koumaki et al. (2021)
Chemical Treatment				
Ozonation		High removal performance	Energy --demanding	Bilińska et al. (2019)
		Simultaneous disinfection and sterilization	Creation of oxidative byproducts	Rueda-Marquez et al. (2020)
Photocatalysis		Ability to remove persistent organic contaminants	Not applicable for many types of wastewaters	Rueda-Marquez et al. (2020)
			Catalyst reusability is a problem	

Among microorganisms that potentially can be implemented in biological treatment, fungal systems have been mostly studied due to their significant ability to degrade ECs (Viancelli et al., 2020). Another advantage of fungal treatment is the flexibility in carbon or energy sources due to the fact that EC removal is essentially the result of the secondary metabolic action of fungi (Harms et al., 2011; Touahar et al., 2014).

Among different types of fungi utilized in ECs removal, white-rot fungi (WRF) and their oxidative enzymes have been mostly reported. Due to being non-specific, ligninolytic enzymes including laccase (Lac; EC 1.10.3.2), manganese peroxidase (MnP; EC 1.11.1.13), versatile peroxidase (VP; EC 1.11.1.16), and lignin peroxidase (LiP; EC 1.11.1.14) secreted by WRF have shown great ability to transform numerous compounds through an oxidation process (Bilal et al., 2019a). Even though each of these enzymes has its specific realm of catalytic action, the principal outcome of the reaction is to produce free radicals and ions in the medium and degrade chemical compounds such as dyes, pharmaceuticals and pesticides (Zdarta et al., 2018a). Among these enzymes, laccase has shown a significant capability of chemical compound transformation and has become a strong potential candidate in wastewater treatment applications (Unuofin et al., 2019).

**FIGURE 2:**
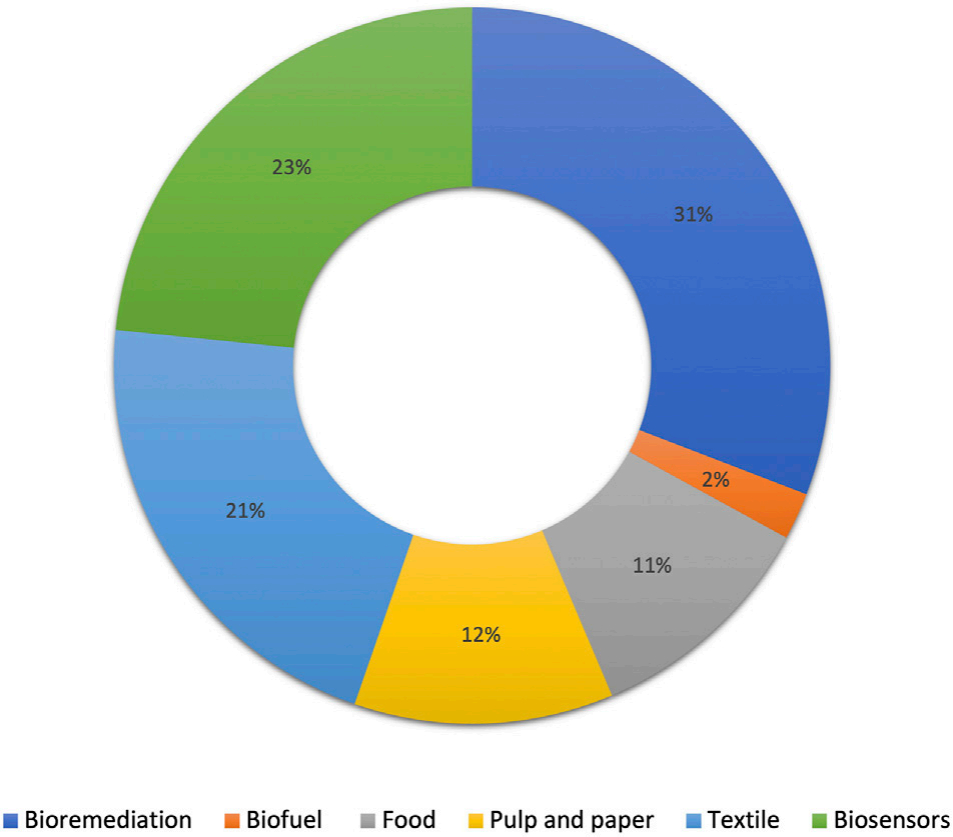
Percentage of laccase application in different industries (adapted from Mate and Alcalde 2017).

Laccases are identified as a group of multicopper oxidases that are widely distributed in plants, bacteria and fungi (Senthivelan et al., 2016). Natural lignin degradation ability is the key feature of laccases; however, thanks to its low-substrate specificity, this enzyme could be implemented in different industries such as biofuel production, bioremediation, pulp and paper, food processing, biosensors, and dye decolorization (Mate and Alcalde, 2017; Antecka et al., 2021). A major application of this enzyme is in the bioremediation area as laccase could oxidize different pollutants such as phenolics, non-phenolics, aromatics, non-aromatics, and carbohydrates (Antecka et al., 2021). Through oxidation, laccase transforms contaminants into smaller components or into high molecular weight oligomers with the concomitant reduction of oxygen molecules into water (Arregui et al., 2019). Figure 2 presents the different percentages of laccase application in different industries.

Although laccase’s ability to eliminate a wide range of contaminants has propelled this enzyme to become a potential candidate for wastewater treatment applications, there are some obstacles regarding its industrial usage, including high production costs, low stability of the enzyme, and its recovery (Hafid et al., 2021). All of these factors directly influence the economic sustainability of such processes. Large-scale production of laccase for industrial application requires a multistep process which can be expensive (Antecka et al., 2021; Hafid et al., 2021). In addition, laccases are generally secreted during fungal secondary metabolism and, unfortunately, the amount of produced laccase from its host is not generally considered sufficient for industrial applications (Antecka et al., 2021; Hafid et al., 2021). A common approach to minimize laccase production cost is to optimize fermentation (process) conditions and reduce the cost of the growth medium (Olivieri et al., 2006). Usually, laccase is produced by fungi grown in single-cell mode in liquid culture. However, through solid-state fermentation laccase could demonstrate higher productivity (Galhaup and Haltrich, 2001). For instance, Xu et al. (2020) reported the noteworthy enhancement in laccase activity secreted from *Trametes versicolor* cultured through solid-state fermentation on tea residue (Xu et al., 2020). Since laccase production efficiency is greatly dependent on growth medium composition (Antecka et al., 2021), the latter can also be optimized towards lower production cost. *Myrothecium roridum* laccase production was significantly increased when hay and rapeseed press cake extract were implemented as carbon sources (Jasińska et al., 2019).

Laccase structure could be distorted and deactivated through changing reaction conditions (Yavaşer and Karagözler, 2021). Moreover, there is no documented industrial application of free laccase (Zerva et al., 2019) due to low stability, poor reusability, and high costs. Laccase immobilization can be used to deal practically with its low stability and recovery. Laccase immobilization over solid supports could crucially increase stability and enable its reuse (Zhang et al., 2021) which, in turn, can contribute to cost reduction of the overall process. For instance, laccase immobilized over rice straw biochar showed increased stability (Imam et al., 2021): after six cycles of usage, immobilized laccase still maintained 47% of its initial activity. Overall, immobilization of laccase on solid supports can increase its stability and reusability along with boosting its activity. However, the efficiency depends upon the methods of immobilization employed. Moreover, the immobilized laccase properties such as immobilization yields, residual activity, subtrate specificity and kinetic parameters depend upon the immobilization methods and supports used (Patel et al., 2016; Patel et al., 2018; Patel et al., 2019).

Various solid supports have been used for immobilization of laccase including materials of various origin and chemical composition such as silica and inorganic materials (Girelli et al., 2020), chitosan (Bilal et al., 2019b), and metal oxides (Zdarta et al., 2018a). The extent of laccase immobilization on these solid supports depends upon their properties such as chemical composition, surface area and functional groups on the surface (Zdarta et al., 2018a). Among these divese supports, carbon based materials have been considered as an ideal candidate for enzyme immobilization (Zdarta et al., 2018a). Carbon-based materials such as activated carbons, graphene, and biochars have been employed efficiently for enzyme immobilization (Zhou et al., 2021). Due to well developed pore structures, high surface area (up to 1000 m^2^ g^−1^), existence of numerous functional groups on the surface, these materials are a valuable candidate for laccase immobilization (Zdarta et al., 2018a).

Among carbon-based materials, biochar, due to its properties, has attracted special attention (Madadi and Bester, 2021). Biochar is a solid carbonaceous material produced through hydrothermal and thermochemical methods (Madadi and Bester, 2021). Biochar is made up of numerous polyaromatic carbon units which enable this material to remove organic and inorganic pollutants from wastewater (Xiang et al., 2020). Further, biochar’s low cost and reasonable adsorption capacity make it a potential candidate for laccase immobilization (Madadi and Bester, 2021). Moreover, biochar has already proved its compatibility with a carbon negative, circular and sustainable economy (Glaser et al., 2009; Bolognesi et al., 2021).

In recent decades, a number of studies developed different immobilized laccase systems to eliminate ECs from wastewater systems. This review is focusing on carbonaceous materials and their role as a growth support for WRF as well as a solid support for laccase immobilization. Moreover, this review highlights the properties of various carbonaceous materials, recent trends in laccase production, and various strategies/mechanisms used for laccase immobilization. It also analyzes and presents the significant benefits of carbon-based materials for their key role in laccase production and immobilization. Furthermore, this review aims to eliminate current research gaps on the immobilization of laccase on carbonaceous materials and provide insights on future research directions in this domain.

## Laccases

### Lignin and Laccases

Lignin is an irregular branched three-dimensional polyphenolic biopolymer, which contributes to plant cell wall structural integrity and stability, resulting in the overall strength and rigidity of woody plants (Joffres et al., 2013; Figueiredo et al., 2018; Bugg et al., 2020). Its complex chemical structure consisting of three basic phenylpropanolic monomers (monolignols), *i.e*., coniferyl, *p*-coumaryl, and sinapyl alcohols makes lignin a highly resistant compound (Joffres et al., 2013; Bagewadi et al., 2017; Figueiredo et al., 2018). Besides, the presence of functional groups such as phenolic hydroxyl, benzylic hydroxyl and carbonyl moieties linked to the monolignols adds to this macromolecule’s heterogeneity and complexity (Bagewadi et al., 2017).

Laccases are one of the best characterized classes of extracellular lignin modifying enzymes (LME) (Zerva et al., 2017; Elisashvili et al., 2018). Owing to their capacity to depolymerize/degrade lignin, laccases attract biotechnological interest as one of the promising “green” tools for phenolic and non-phenolic compounds transformation and environmental bioremediation (Kameshwar and Qin, 2017; Zerva et al., 2017). Laccases are naturally expressed in bacteria, plants, or fungi (Kameshwar and Qin, 2017). WRF species, which play a major role in the wood decay process, are under considerable scrutiny in research for LME production (Ergun and Urek, 2017; Elisashvili et al., 2018).

Enzyme production is an important field in biotechnology. Given the promising biotechnological and industrial applications of laccases, continuous efforts have been deployed for the optimization of their production, aiming at their catalytic property enhancement and minimizing production costs. Bioengineering of new producing fungal species, optimization of the production methods and cultivation media, or bioprocess technologies are the avenues usually exploited (Pollegioni et al., 2015; Elisashvili et al., 2017; Kumar and Chandra, 2020).

### Fermentation Strategies for the Production of Laccases

Typically, submerged (SmF) and solid-state fermentations (SSF) of lignocellulosic materials by WRF are used for laccase production (Elisashvili et al., 2018). SSF involves the growth of microorganisms on solid natural (e.g., organic substrates) or synthetic inert materials in the absence or near absence of free liquid medium (Palma et al., 2016; Ergun and Urek, 2017). This approach offers attractive features such as the use of cheap and underutilized agroforestry wastes as growth substrates to produce high value-added enzymes, high volumetric productivity, low energy and operational cost, low wastewater production, and low susceptibility to bacterial contamination (Karp et al., 2015; Soumya et al., 2016; Ergun and Urek, 2017; Ariste et al., 2020). SSF has been shown to be particularly fitting for filamentous fungi, since it provides adequate surface adherence and tends to mimic their natural habitat and growth conditions (Chenthamarakshan et al., 2017; Soccol et al., 2017).

Under SmF, microorganisms are grown in carbohydrate-based liquid media usually supplemented with nitrogen and other nutrients, under aerobic conditions. Unlike SSF, SmF allows easy monitoring of operating parameters such as pH, dissolved oxygen, or concentration of water-soluble substrates. In addition, this system is characterized by an easy mixing of the broth and separation of the biomass after fermentation. Due to its relatively easy scale-up, industrial production of enzymes is mainly performed under SmF (Wang et al., 2019). However, SmF can be limited by uncontrolled mycelial growth resulting in an overabundant biomass. Expansion of biomass can increase broth viscosity and limit mass and oxygen transfer, thereby reducing metabolic rate and enzyme secretion (Krull et al., 2013; Silvério et al., 2013).

### Co-culture: An Effective Strategy for the Enhanced Production of Laccase

In recent years, microbial co-culture has developed rapidly as a promising alternative for the biosynthesis of various natural bioproducts of interest (Qian et al., 2020). This technique, which can be performed under SSF or SmF, brings together different species. It is therefore a convenient way to exploit the interactions of different species and stimulate individual strain cryptic genes and trigger the generation of new products. Yet, the exact biosynthetic mechanisms and pathways behind the overall process are complex and still await elucidation (Maglangit et al., 2020; Zhuang and Zhang, 2021). To be successful, biosynthesis of new products in co-culture requires appropriate conditions for the compatible coexistence of the different microbial species involved (Zhuang and Zhang, 2021). In terms of compatibility, different interactions have been highlighted between species in co-culture fermentations: one species develops at the expense of the others, the species inhibit each other (deadlock), or they collaborate (Wiberth et al., 2019).

Several recent studies on co-culture have proven its feasibility and viability as an experimental approach to enhance the chemical diversity of microorganisms. Co-culture of *Pycnoporus sanguineus* and *Beauveria brongniartii* strains under SSF by Jiménez-Barrera et al. (2018) yielded a six-fold increase in laccase activity. Also, a co-culture of *Pycnoporus sanguineus* and *Trametes maxima* and eight soil-borne micromycetes under SmF showed different competitive antagonism and collaboration interactions while, overall, ligninolytic enzymes including laccase showed increased activity (Wiberth et al., 2019). Laccase enzyme systems have been produced by co-cultures of *Alcaligenes faecalis */* P. sanguineus* (Li et al., 2016) and *T. maxima* /* Paecilomyces carneus* (Chan-Cupul et al., 2016) under SmF; both yielded higher laccase activity compared to monocultures.

### Factors Affecting Laccase Production Under SmF and SSF

Under solid-state or submerged fermentation, several factors can influence enzyme production. Successful production implies selection of appropriate fungi species, supports/substrates, growth media and conditions, and inducers (Soccol et al., 2017). In general, the key factors that regulate laccase production can be clustered into two broad sets. The first category includes the media composition (in particular the carbon and nitrogen sources and concentrations), the concentration of dissolved oxygen (DO) and the type and concentration of inducers (Kumar et al., 2016; Elisashvili et al., 2017; Schneider et al., 2020). Second, the operating parameters, which comprise pH, temperature, agitation, and incubation time can significantly affect fungal laccase production. As the effects of these factors combine, it is quite complex to establish a standardized model for the regulation of laccase synthesis (Chenthamarakshan et al., 2017; Elisashvili et al., 2018).

#### Importance of Carbon and Nitrogen Sources on Laccase Production

As a first note, different fungi may require different sources of carbon and nitrogen to fully release their laccase expression potential. Under submerged conditions, Hariharan and Nambisan (2012) tested many sources of carbon including glucose, sucrose, starch, maltose, and lactose. Their results suggested that glucose and sucrose enhanced the enzyme expression, but other carbon sources contributed to activity decrease. These results are consistent with those recently unveiled by other researchers, where glucose effectively promoted laccase activity (Schneider et al., 2019; Marin et al., 2020). Furthermore, Schneider et al. (2019) found that the secretion of laccase was related to the nitrogen source in the media, with casein being a better enzyme promoter than peptone. In the same sense, *Lentinus strigosus* 1566 showed highest laccase activity in a peptone-yeast extract medium supplemented with galactose, arabinose, and xylose, while glucose, sucrose, or maltose decreased its activity (Myasoedova et al., 2015). These authors also found that glucose slightly increased laccase activity compared to malt dextrin, whereas fructose decreased laccase production. As for sucrose and glycerol, they lowered laccase activity yield but substitution by maltose had no effects on laccase production. Overall, diverse carbon sources have a significant role in laccase production. Determining the best carbon source is the first step towards optimal growth medium design and eventually optimal laccase production.

Typically, culture media are supplemented with organic or inorganic nitrogen sources. Depending upon these two forms, different levels of laccase expression and activity can be observed with the same strain and from one strain to another. A direct positive correlation between peptone concentration and biomass development and laccase activity increase was observed in a culture of *Coriolopsis gallica* 142 strain (Mikiashvili et al., 2006; Elisashvili et al., 2017). However, at a certain threshold, the subsequent increase in peptone concentration led to an opposite effect on the activity. In the aforementioned study, nitrogen sources such as peptone, yeast extract, beef extract, ammonium sulphate, ammonium nitrate, and urea, were also tested for laccase production. The authors found that beef extract was the best nitrogen source for highest activity expression after 120 h of incubation (Hariharan and Nambisan, 2012). Previously, Zerva et al. (2017) studied the comparative influence of five different nitrogen sources including diammonium tartrate, potassium nitrate, ammonium nitrate, yeast extract and corn steep liquor (CSL) on laccase expression by *Pleurotus citrinopileatus* and *Irpex lacteus*. It was observed that both species developed highest biomass and laccase activities in samples supplemented with CSL. Besides, inorganic nitrogen sources were found to promote less fungal growth. In another study, Chauhan (2019) obtained a similar result with *Grammothele fuligo* cultured in glucose-based medium, where inorganic nitrogen sources tested failed to promote abundant biomass and further to secrete laccase. The fermentation of *P. ostreatus* Pl 22 strain using different nitrogen sources showed that yeast extract increased laccase activity by almost six-fold in comparison with ammonium sulfate (Karp et al., 2015). Mikiashvili et al. (2006) determined that ammonium sulfate and ammonium nitrate were good sources of nitrogen for laccase production by *Trametes multicolor*. Besides their individual effects, the Carbon/Nitrogen (C/N) ratio can significantly influence the synthesis and secretion of fungal laccase (Rivera-Hoyos et al., 2013; Elisashvili et al., 2018). Globally, depending upon the strains, low or high C/N ratio can alternately improve or decrease the production (Elisashvili et al., 2018). Interestingly, Yang et al. (2016) determined that the combination of high concentrations of carbon and nitrogen led to higher production of laccase from *Cerrena* sp.

In summary, a wide range of nitrogen sources has been studied and can induce diverse effects on laccase production, hence there is considerable uncertainty regarding the selection of the optimal nitrogen concentration for laccase production (Elisashvili et al., 2018).

#### Effect of Inducers on Laccase Production

Lignin degradation metabolites and metals naturally present in the environment can act as promoters of fungal laccase production. In a laboratory context, phenolic and aromatic compounds, especially those structurally related to lignin (Furukawa et al., 2014; Pollegioni et al., 2015; Elisashvili et al., 2017), and metals such as copper, manganese, cadmium, and magnesium can play an important role in laccase production (Valle et al., 2014; Martani et al., 2017; Lallawmsanga et al., 2019). However, these compounds have also been depicted to be playing dual roles as they can act as inducer or repressor, depending notably on their concentration, the media composition, the fungal species, and the enzyme tested (Elisashvili et al., 2017). Under submerged fermentation, hydroquinone was found to cause an increase in laccase production by *T. versicolor*, whereas *C. unicolor* rather decreased laccase activity (Elisashvili et al., 2010). Under laboratory conditions, compounds such as 2,5-xylidine, guaiacol, veratryl alcohol (VA) and catechol are often used as laccase inducers (Krull et al., 2013; Martani et al., 2017). In a submerged fermentation of *T. multicolor* 511, VA and guaiacol enhanced laccase specific activity by two-fold (Mikiashvili et al., 2006). Similarly, gallic acid (1 mM), tartaric acid (20 mM), and citric acid (20 mM) could elevate laccase activity (Chang and Chang, 2016). It was also observed that among several organic inducers, ethanol and guaiacol induced laccase production by *Lentinus crinitus* while pyrogallol, veratryl alcohol, xylidine, and vanillin were ineffective (Valle et al., 2014)*.* It was also determined that the induction of laccase activity by ethanol was concentration-dependent, as concentrations of 1% v/v and 3% v/v have increased *Ganoderma lucidum* laccase activity production by 6.5 and 14 times compared to the control, repectively. However, with up to 5% v/v ethanol, the activity reached only 10 times that of the control, showing that the correlation of activity induction with ethanol concentration was positive up to a certain level, beyond which the ethanol concentration could be less effective in increasing laccase activity (Manavalan et al., 2013). Resveratrol, tannic acid, and guaiacol were found to be the best laccase inducers in a culture of *C. gallica*, however, 2–2′-azinobis (3-ethylbenzothiazoline-6-sulfonic acid) (ABTS) and gallic were ineffective (Xu et al., 2016) under the same fermentation conditions. On the contrary, laccase activity was increased in *Cerrena* sp. HYB07 fermentation by ABTS and guaiacol, though other aromatic compounds had no significant effects (Yang et al., 2016).

Several inorganics can modulate laccase expression. In general, trace metallic elements at high concentrations can be toxic to ligninolytic fungi growth and repress their laccase expression. Besides, it was demonstrated early on that tolerance to high concentrations of trace metallic elements can largely be species dependent (Giller et al., 1998; Valle et al., 2014). Meanwhile, some metallic compounds such as Cu, Mn, Co, and Zn, present at low concentrations in the culture medium are essential for fungal growth and biological functions (Baldrian, 2003; Asif et al., 2017). Among microelements, copper is largely used as an inducer in enzyme production. The positive correlation between laccase production and copper, often added to the media as copper sulfate, has been well described in previous studies (Valle et al., 2014; Karp et al., 2015; Chang and Chang, 2016; Vrsanska et al., 2016; Yang et al., 2016; Zhu et al., 2016; Schneider et al., 2019). Moreover, the influence of copper on laccase expression is likely to be magnified or minimized concomitantly with high or low nitrogen concentration, respectively (Valle et al., 2014). However, under certain conditions, the negative effect of copper has also been highlighted (Dao et al., 2019). Thus, as reported by Martani et al. (2017), the overall influence of copper on laccase production depends on its concentration in the culture medium, the microbial strains involved, and the presence of other components in the medium.

#### Effect of Fermentation Operating Parameters on Laccase Production

In addition to the design of the nutritional environment, operational factors such as temperature, pH, time, agitation rate, and dissolved oxygen can significantly influence the fungal growth and enzyme production.

Temperature does not correlate significantly with fungal growth rate and biomass development (Martani et al., 2017). However, it importantly influences the potential and the level of laccase activity expressed, as revealed by several studies. Schneider et al. (2019) showed that laccase activity of *Marasmiellus palmivorus* VE111 was maximum at 28°C and decreased when this temperature was either lowered or raised by 5°C. The decrease of laccase activity below or above 28°C was explained by the reduction of expression of some genes involved in the transcription of this enzyme (Rivera-Hoyos et al., 2013; Schneider et al., 2019). A previous study on *M. palmivorus* LA1 laccase secretion under SSF using pineapple leaf as substrate led to a similar conclusion (Chenthamarakshan et al., 2017). Hariharan and Nambisan (2012) found that 27°C was the best temperature for laccase production by *Ganoderma lucidum* under SSF, while temperatures lower than 23°C or higher than 33°C led to a significant reduction in enzyme production. Yet, Chang and Chang (2016) determined 30°C as the optimum temperature for laccase production from *Pleurotus eryngii*, under submerged conditions.

The pH can have an important influence on fungal growth and thereby on laccase expression. According to previous studies, highly acidic or basic media negatively affect fungal growth and laccase activity, and this can be noticed either under SSF or SmF. Chang and Chang (2016) noted an increase of laccase activity of *P. eryngii* between pH 2 and 5, before its decrease in the 5–9 pH-range. In another study, Chenthamarakshan et al. (2017) determined that pH 5 was the optimum for best growth of *M. palmivorus* LA1 on pineapple leaf for laccase secretion and maximum activity. In the same vein, pH 5 was determined as optimal for production of laccase from *G. lucidum* under SSF, after an optimization process (Hariharan and Nambisan, 2012) while Zerva et al. (2017) got the best results at pH 5 and 6 with *Pleurotus citrinopileatus* and *Irpex lacteus* strains using supplemented olive mill wastewater as culture medium. For Schneider et al. (2019), pH 4 and below or pH 8 and above led to laccase activity decrease, whereas it reached maximum activity at pH 7.

Incubation time for an enzyme to reach maximum activity expression varies from one strain to another and according to fermentation conditions. In general, microorganisms are characterized by a period of acclimation followed by growth and biomass production accompanying the substrate consumption. Overall, thanks to the ready availability of nutrients, the culture period for enzyme production in SmF is generally shorter than that of SSF (Wang et al., 2019). The *Ganoderma lucidum* 447 culture for enzyme production in olive mill by-products medium achieved highest laccase activity after 6 days, *i.e.* earlier than with other fungi tested in the same study. In contrast, *Cerrena unicolor* 302 attained maximum laccase activity after 2 weeks of fermentation (Elisashvili et al., 2017). A 2-week period was also the cultivation time necessary for *Ganoderma applanatum* with rice bran as media to achieve maximal laccase activity (Wang et al., 2019). The culture of *Coriolus versicolor* on sweet sorghum bagasse in SSF supplemented with CuSO_4_, gallic acid and syringic acid produced maximum laccase activity within 16 days (Mishra et al., 2017). Under SmF, *P. citrinopileatus* and *I. lacteus* produced highest laccase activity in 10 and 24 days of cultivation in olive mil wastewater, respectively (Zerva et al., 2017), however *Cerrena consors* took much more time (30 days) for the laccase activity peak in a 50% olive mill wastewater (Mann et al., 2015).

Under submerged fermentation conditions, the availability and transfer of oxygen is essential for fungal growth. As mentioned earlier, mycelial uncontrolled expansion can limit oxygen transfer (Krull et al., 2013; Silvério et al., 2013). To promote oxygen transfer, it is important that the culture must remain continuously under shaking conditions. This was corroborated by Domingos et al. (2017) who found that unshaken culture resulted in incomplete sugar consumption partially due to lack of proper oxygen transfer. In another study, Schneider et al. (2019) have analyzed the influence of the concentration of dissolved oxygen on enzymatic activity from *Marasmiellus palmivorus* VE111 strain. Thus, in general, it is proved that increased laccase activity is directly related to DO concentration.

The monitoring of agitation has shown a positive correlation between biomass growth and agitation rate. However, above a certain threshold, agitation can lead to a negative effect on biomass growth and enzyme expression. In fact, under excessive agitation, hydrodynamic shear stress on biomass can result in changes in its morphology, leading to subsequent enzyme under-expression (Zerva et al., 2017).

### Sustainable and Cost-Effective Growth Media for Enhanced Production of Laccases

Recently, several fungal strains have been screened for their potential growth under SmF conditions for laccase production, using various natural carbonaceous substrates such as agro-residues. For instance, Elisashvili et al. (2018) used mandarin peels (MDP), olive tree sawdust (OTS), olive pomace (OP), and olive mill wastewater (OMW) as growth substrates under SmF and SSF conditions. They have tested seven strains belonging to *C. unicolor*, *Fomes fomentarius*, *Ganoderma lucidum*, *P. ostreatus*, *P. coccineus*, *T. trogii*, and *T. versicolor* species*.* The culture media were initially supplemented with 0.3% peptone as additional nitrogen source and 1 mM CuSO_4_ as laccase inducer. Overall, *C. unicolor* and *T. trogii 146* strains showed the highest laccase activity. MDP were good substrates for laccase secretion by the *C. unicolor* strains, and OTS promoted best secretion of laccase by *C. unicolor* 302, whereas OP appeared to be ideal for laccase production by *C. unicolor* strains and *T. versicolor* (Elisashvili et al., 2018). Cultures with OMW favored enhanced production of laccase by G*. lucidum* 447, *P. ostreatus* 2175, and P. *coccineus* 310. Overall, highest laccase activity was obtained from *C. unicolor* 301 and *T. trogii* 146 with OMW-based medium. In a similar study, Zhao S.-X. et al. (2017) grew *P. ostreatus* under SmF conditions using tea, peanut shells, orange peel, corn cob, and bagasse as substrates in glucose-based medium. Laccase production was enhanced in all the cultures except in those using peanut shells as substrates. The cultures with orange peel showed the highest laccase activity which was nine times higher than the control.

## Immobilization of Laccase

**FIGURE 3:**
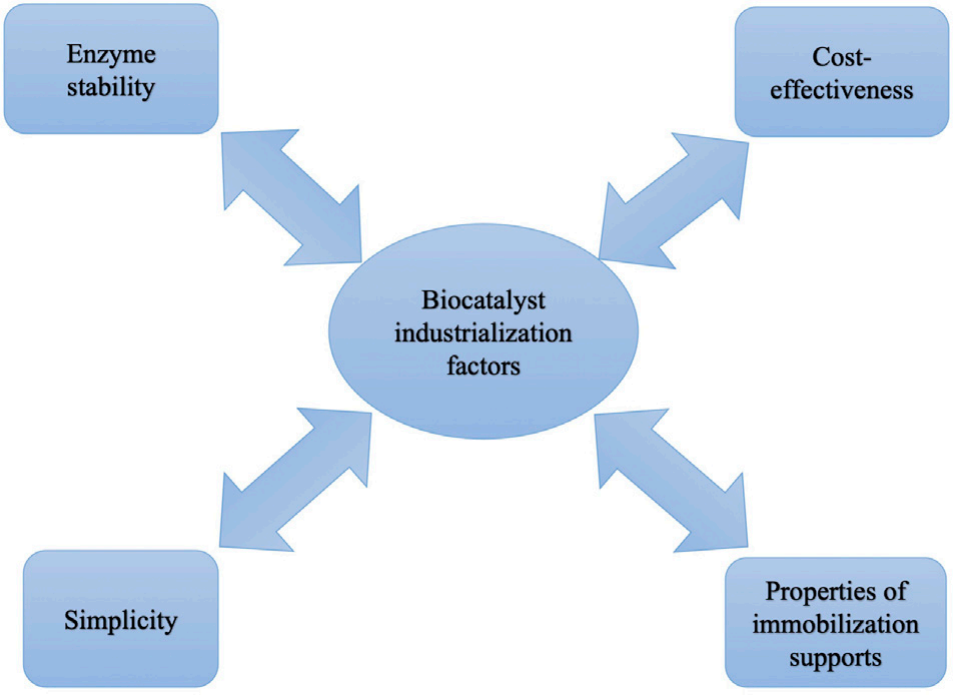
Important factors to be considered in biocatalyst design and synthesis.

Free laccase can have high activity. However, due to not being able to separate and be reused, activity can be lost in a continuous process thus increasing the operational cost (Masjoudi et al., 2021). In addition, it has been proved that free laccase may exhibit poor stability while exposed to harsh operating conditions and over time (Wen et al., 2019). In order to tackle these challenges, the immobilization strategy is considered the most successful method. Attachment of laccase over solid supports could significantly enhance its capability to maintain its activity over time and its resistance to operational conditions (e.g. temperature, pH, and exposure to different chemical agents) (Shakerian et al., 2020). Moreover, reusability of immobilized laccase can crucially decrease operational cost in continuous systems (Naghdi et al., 2017). However, immobilization could result in laccase conformational change, and a decrease in activity (Ji et al., 2017). For an efficient immobilization, mode of immobilization, support material, and initial activity of laccase are critical parameters to be considered (Yavaşer and Karagözler, 2021). Figure 3 shows the important factors regarding biocatalyst preparation.

### Modes of Immobilization

Immobilization procedures are categorized into two groups including physical and chemical interactions (Fernández-Fernández et al., 2013). The difference between chemical and physical immobilization procedure refers to how the enzyme attaches onto/into the support (Zdarta et al., 2018a). During physical immobilization, there is no or minimal enzyme conformation change, and the enzyme could keep its activity (Zhou et al., 2021). In this methodology, there are no strong interactions between enzyme and carrier and the two can be connected through weak intermolecular forces such as hydrogen bonds, ionic, and hydrophobic interactions (Ba et al., 2013; Zdarta et al., 2018b). Entrapment and adsorption stand out as the main physical procedures (Zhou et al., 2021).

In contrast to physical attachment, chemical interactions are involved through the creation of covalent bonds between enzyme and solid support (Daronch et al., 2020). Chemical immobilization is based on the interaction between functional groups of the solid support and enzyme functional groups (mostly –NH_2_, –SH, and –OH). Covalent binding and cross-linking can be considered as two methodologies in this category.

**FIGURE 4:**
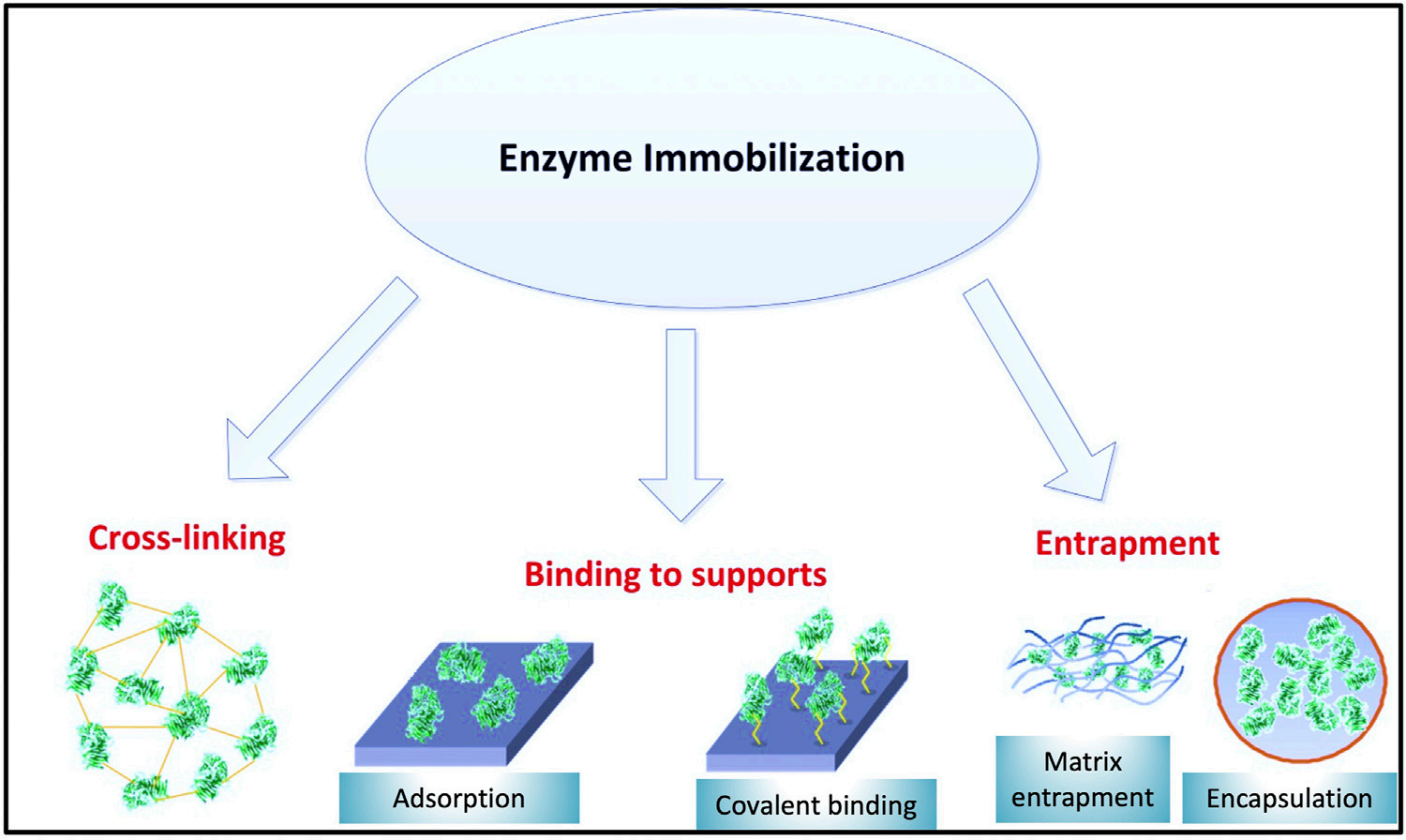
Enzyme immobilization methods.

Since physical bonding is relatively weak, it will maintain the enzyme bound to the support for a shorter period of time (Datta et al., 2013). In addition, changes in operational conditions (e.g. ionic strength, pH, and temperature) could result in loss of enzyme activity. As a result, preference is given to chemical immobilization (Wahab et al., 2020) for industrial applications such as wastewater treatment. Generally, it is expected that chemical immobilization reduces enzyme leakage and significantly improves its reusability (Zdarta et al., 2018b). Figure 4 illustrates different immobilization techniques.

#### Entrapment

Entrapment is identified as the simplest immobilization technique in which enzyme molecules disperse into a porous solid matrix; hence no direct attachment may be formed between carrier and enzyme (Fernández-Fernández et al., 2013; Karthik et al., 2021). Alginate, collagen, silicon rubber, gelatin, carrageenan, polyurethane, polyacrylamide, and polyvinyl alcohol with styryl pyridinium groups are solid matrices that can be used for enzyme entrapment (Dayaram and Dasgupta, 2008; Phetsom et al., 2009; Fernández-Fernández et al., 2013). Enzyme entrapment can be carried out in two steps: first enzyme molecules are dispersed into monomer solution, and then a polymerization process ensues which maintains enzyme molecules trapped (Karthik et al., 2021). Entrapment technology could increase laccase stability considerably and it can be helpful to avoid enzyme denaturation. Despite its benefits, this method has some limitations which restrict its application. One such issue is enzyme leakage which can be significant when a support with a large pore size is used.

#### Adsorption

In the adsorption immobilization technique, the enzyme is linked to the carrier through weak interactions (Sirisha et al., 2016). Based on the types of weak forces, adsorption immobilization can be divided into two categories, namely ionic attachment (electrostatic interaction is dominant) and physical attachment (mainly through van der Waals forces, hydrophobic interactions or hydrogen bond formation) (Karthik et al., 2021; Zhou et al., 2021). Compared with other techniques, adsorption methodology is recognized as a simple and low-cost procedure for enzyme immobilization (Fernández-Fernández et al., 2013). Despite its benefits, the amount of enzyme leakage in this method is high, therefore the application of adsorption immobilization for long-term processes or processes with varying operational conditions is not recommended (Zhou et al., 2021). pH, ionic strength of the solution and solid support surface area are three factors that should be considered during adsorption immobilization (Rekuć et al., 2008; Huajun et al., 2009; Xu et al., 2009; Forde et al., 2010).

#### Covalent Binding

Covalent binding is considered as the most reliable method for industrial application (Fernández-Fernández et al., 2013). In this methodology, strong bonds are formed between non-essential amino acids at the surface of enzymes and carrier chemical groups. Due to the formation of these strong bonds between supports and enzymes, the amount of leakage decreases significantly (Hernandez and Fernandez-Lafuente, 2011; Zdarta et al., 2018b). Based on the functional groups on the supports, various reagents could be implemented to prepare the support for covalent immobilization. For supports with hydroxyl groups, cyanogen bromide (CNBr) and carbonyl diimidazole (CDI) are recommended (Karthik et al., 2021). For supports with carboxyl groups, zero length reagents such as EDC (1-ethyl-3-(3-dimethylaminopropyl) carbodiimide hydrochloride), NHS (N-hydroxysulfosuccinimide), and EDC coupling with Sulfo-NHS are recommended (Hermanson, 2013). In addition to these reagents, ionic liquids have been frequently used in enzyme immobilization as they are eco-friendly solvent media (Hermanson, 2013). However, selection of ionic liquid types is a key step since cation or anion changes in such a liquid could affect activity, structure and enzyme stability (Hermanson, 2013). The possibility of laccase immobilization on magnetic nanoparticles was also investigated (Qiu et al., 2020). In this study, the surface of magnetic nanoparticles was modified with an amino-functionalized ionic liquid. Through surface modification with 3-(chloropropyl) trimethoxysilane (CPTMO) and (3-aminopropyl) trimethoxysilane (APTES), laccase was covalently immobilized on the surface (Qiu et al., 2020). Stability-wise, the biocatalyst could maintain around 70% of its initial activity after six cycles (Qiu et al., 2020). In the context of magnetic supports, bioinspired magnetic particles bearing laccase (laccase-biotitania, lac-bioTiO_2_) were applied for the efficient removal of bisphenol A, 17α-ethinylestradiol and diclofenac in a mixture of six model endocrine disrupting compounds (EDCs) and retained 90% of activity after five reaction cycles and 60% after 10 cycles (Ardao et al., 2015).

#### Cross-Linking of Enzyme Aggregates

Cross-linking of enzyme aggregates is a carrier-free insolubilization procedure in which multifunctional or bifunctional reagents are implemented to assist enzyme cross-linking into a unified structure with no added carriers (Mateo et al., 2004; Zhou et al., 2021). Since, in this methodology, enzymes act as their own solid supports, this procedure is also called a self-immobilization technique (Karthik et al., 2021). Among different cross-linker reagents such as diiminoesters, diisocyanates, and diamines activated by carbodiimide, the best-known is glutaraldehyde (GA) as it is inexpensive, widely available, and easy to manipulate (Fernández-Fernández et al., 2013; Xiang et al., 2018). However, currently this cross-linker is raising potential toxicity concerns (Yang et al., 2019). This method is highly dependent on pH which includes Schiff’s base formation and Michael-type 1,4 in addition to α, β-unsaturated aldehyde moieties (Migneault et al., 2004). There are two kinds of enzyme cross-linking techniques, namely formation of cross-linking enzyme crystals (CLEC), and of cross-linking aggregates (CLEA) (Ba et al., 2013). In CLEA (Schoevaart et al. 2004), first enzyme molecules are clustered in chemical precipitant solutions such as acetone, ammonium sulfate or ethanol and subsequently a cross-linking reaction completes the process, as initially demonstrated with laccase CLEA by Cabana et al. (2007) and then by others (Matijošyte et al., 2010; Nguyen et al., 2017). CLEC techniques demonstrate good stability and promising activity, however for this process high purity of enzyme is required (Sirisha et al., 2016). Finally, cross-linking with the concomitant enzyme immobilization on an inert porous support may confer additional stability. For istance, Nair et al. (2013) described the deactivation of free and immobilized enzymes during their incubation at 45, 55, 65 and 75°C at pH 5 in absence of electron-donor substrate by periodically measuring the residual activity with ABTS as a substrate. An apparent higher stability of immobilized laccase was evidenced with greater half-lives for the immobilized laccase than soluble laccase. Table 2 presents indicative properties of enzyme immobilization techniques applicable to laccases.

**TABLE 2:** Inherent characteristics of immobilization methods (Zhou et al., 2021).

Characteristics	Entrapment	Adsorption	Covalent binding	Self-immobilization
Cost	+	++	+++	+
Preparation difficulty	+	+	+++	++
Stability	+	+	+++	+
Binding force	+	+	+++	+++
Enzyme leakage	+++	++	-	-
Diffusion resistance	++	-	+++	-
Laccase protection	++	-	-	-
Activity loss	+	++	+++	+++
Applicability	++	++	+++	+

### Immobilization Carriers/Solid Supports and Their Properties on Laccase Immobilization

**FIGURE 5:**
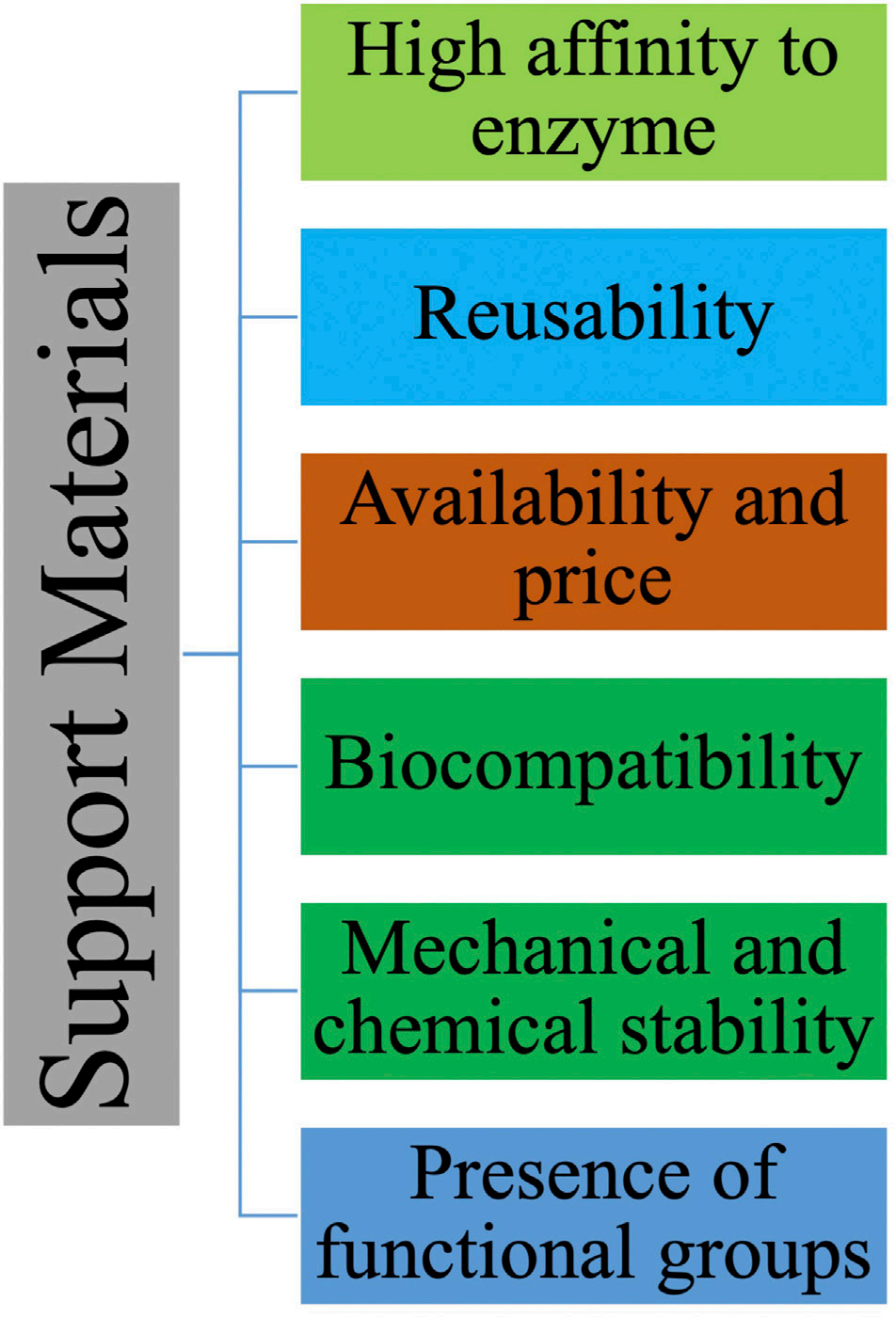
Support properties for laccase immobilization.

The selection of appropriate solid support for laccase immobilization is crucial for biocatalyst efficiency (Daronch et al., 2020). Generally, carriers are sought to enhance laccase catalytic activity and stability (Zhou et al., 2021). An ideal support should protect both enzyme structure and activity under a variety of operational conditions (Zdarta et al., 2018a) while keeping its own physical integrity. Here below, important characteristics of a solid support are discussed (Figure 5).

#### Particle Size

Solid support particle size plays a significant role in the success of immobilization. In industrial applications, large particles may be handled better than small ones (Santos et al., 2015). Nanoporous gold supports were employed to study the effect of particle size on laccase immobilization (Huajun et al., 2009). The results obtained from three different particle size samples demonstrated that the larger particle size support had the ability to keep more enzyme on its surface due to laccase accessibility to inner pore structures (Huajun et al., 2009). However, having larger support particles could have some drawbacks as well. Large particles could enhance diffusional limitations which could, in turn, affect negatively the enzyme activity (Bortone et al., 2014). In the case of the substrate, if its consumption rate by the enzyme is higher than its diffusion rate, there is a possibility of the enzyme located at the support core not receiving any substrate and therefore the biocatalyst’s apparent enzyme activity could decrease (Lortie and André, 1991; Boniello et al., 2010). At the same time, even though nanoparticles present handling issues, the diffusion problems can be prevented by the use of nanoparticles instead of microparticles and for non-porous supports the enzyme is always exposed to the substrate (Bilal and Iqbal, 2019; Bilal et al., 2020). Moreover, to produce effective multipoint covalent immobilization on nanoparticles, epoxy, glyoxyl or divinylsulfone activated nanoparticles can be used (Bilal and Iqbal, 2019; Bilal et al., 2020).

#### Pore Size/Specific Area

There is a connection between pore size and surface area in which larger pores result in a lower specific area. Specific surface area determines the amount of enzyme that could be loaded over the carrier (Di Cosimo et al., 2013). From an economics perspective, a larger specific surface area could result in a higher amount of enzyme that could be loaded over the support (Santos et al., 2015). Pore diameter determines the size of the enzyme which could be immobilized over the solid support. Importantly, the size of the pore should be big enough to allow the new enzyme molecules to enter in the support (Hudson et al., 2008). In general, the diameter of the pore should be four to five fold larger than the enzyme’s molecule size (Hanefeld et al., 2009). According to a comprehensive analysis of 182 experiments with emphasis on the effect of pore size and surface area on enzyme immobilization, a general trend emerged: higher surface area would result in higher enzyme load on the support (Bayne et al., 2013). However, this general trend for pore size was divided into three ranges in which for the supports with pore size less than 10 nm, the amount of loading is less (apparently due to physical restrictions in accessing the augmenting surface inherent in this pore diameter range), for the supports with pore size between 10 and 100 nm, the amount of enzyme loading tends to be constant (possibly due to protein–protein interaction blocking pores and restricting access to the higher surface area available at lower pore diameters), and for supports with pore size higher than 100 nm, the amount of enzyme loading per unit mass would decline due to a parallel reduction in available surface area (Bayne et al., 2013). Thus, upon a critical analysis even if the surface area is larger for solid supports with small pores the possibility of enzyme loading is lower. Moreover, there was no clear trend between pore characteristics and retention of catalytic activity (Bayne et al., 2013).

#### Functional Groups

The existence of functional groups on the solid supports is another factor that controls enzyme-support interactions (Santos et al., 2015). Favorable functional groups on the solid support are essential to ensure that strong multiple interactions would occur between enzyme, binding agent, and support leading to decreased leakage (Pandey et al., 2020). While the density of active groups on the solid support is crucial, the nature of functional groups is also critical. Most active groups are stable and do not require further consideration (Garcia-Galan et al., 2011). However, covalent immobilization merits further analysis (Garcia-Galan et al., 2011). An ideal functional group for successful covalent immobilization should have the following properties:

- Allow reaction between enzyme and support with low steric hindrances (Mateo et al., 2005);
-Maintain the physical properties of the enzyme after immobilization (Bolivar et al., 2009);
- Be stable over a wide range of conditions (Pedroche et al., 2007);
- Require a simple immobilization protocol with no additional treatment (Santos et al., 2015).

#### Inertness and Mechanical Properties

Support inertness could affect both immobilization and the substrate on which immobilized laccase is expected to act (Daronch et al., 2020). Commonly, a solid support should maintain its physical integrity and be inert after immobilization to avoid interfering with desired reactions (Ba et al., 2013). Polysaccharide matrices such as agarose and cellulose beads, carbonaceous materials, as well as silica compounds are considered as inert solid supports (Santos et al., 2015). Mechanical properties of solid supports are highly dependent on the process use intended for the immobilized laccase (Garcia-Galan et al., 2011). For instance, in a fixed-bed reactor, the solid support should have high rigidity to tolerate high pressure (Santos et al., 2015), hence silica materials, carbon-based materials, and inorganic oxides are recommended (Kim et al., 2008; Tartaj, 2011; Hartmann and Kostrov, 2013). However, the situation would be different in a stirred-tank reactor (Santos et al., 2015)where, instead of mineral materials, more flexible compounds such as agarose beads, cellulose beads, and lentikats can be used (Grazu et al., 2006; Cárdenas-Fernández et al., 2012; Lam et al., 2012).

Besides the above-mentioned properties, the ideal solid support should be low cost and eco-friendly (not increasing operation cost and generating environmental problems), with high affinity toward the enzyme to be amenable to regeneration (Ba et al., 2013; Daronch et al., 2020). Table 3 categorizes three major types of support materials used for immobilization and their specific properties.

**TABLE 3:** Categories and properties of support materials for immobilization.

Material types	Advantages	Examples
Organic	Presence of functional groups, biocompatibility, abundant in nature	Chitosan, cellulose, agar, synthetic polymers, etc
Inorganic	Good pH and temperature stability, mechanical resistance, operational stability	Silica, alumina, active carbons, biochar, etc
Hybrid and composite	Reusability, strong binding to enzyme, high stability	Alginate-chitosan, silica magnetite, etc

## Carbonaceous Materials in Laccase Production and as a Support for Their Immobilization

### Perspectives of Carbon-Based Materials for Laccase Production As Inducers and Growth Medium

The prospect of using carbon-based materials is very interesting for laccase production. However, there are few reports in the literature on biochar utilization in laccase production, in contrast to more abundant trends towards biochar immobilization of enzymes produced conventionally. Another technique involves the concomitant production and immobilization of enzymes on solid supports in a single-step process. However, to the best of our knowledge, this has not been explored further and future studies can further explore the concerted production and immobilization of enzymes within the same process. Fortunately, due to the eclectic and rich composition of biochar and its overall physicochemical characteristics (see below), the use of this material can be considered a multi-in-one technique to enhance laccase production and immobilization.

#### Biochar as a Substrate for Production and Support for Immobilization

The study of biochar’s composition has revealed that, depending on the feedstocks and pyrolysis conditions, this material can present incompletely degraded lignocellulosic biomass and nitrogen-content residues such amine groups (see below). Furthermore, functionalization can introduce new chemical groups to the biochar structure. These elements make biochar a complementary source among the common carbonaceous nutrients provided in culture media for laccase production WRF. Besides, the large specific area and pore size, and the existence of specific chemical groups on biochar surface favor its adsorptive capacity, which can also be related to the molecular size of the enzyme (Rajapaksha et al., 2016; Li et al., 2018; Fernandez-Sanroman et al., 2020; Pandey et al., 2020). Several studies have reported the successful enhancement of laccase production and immobilization on biochar either by adsorption or covalent bonds (Lonappan et al., 2018a; Li et al., 2018; Fernandez-Sanroman et al., 2020; Pandey et al., 2020; Imam et al., 2021). A summary of such studies is shown in Table 4.

**TABLE 4:** Immobilization of laccase on carbon-based materials.

Source of enzyme	Support	Pre-treatment	Immobilization loading	Relative activity	Re-usability	Reference
*Trametes maxima*	Rice straw	HCl	66%	-	40% (six cycles)	Imam et al. (2021)
*Aspergillus niger*	Commercial activated carbon	No	-		70% (five cycles)	Daoud et al. (2010)
*Aspergillus sp.*	Activated carbon fibers	Dopamine	23%		60% (six cycles)	Zhang et al. (2018a)
*T. versicolor*	Rice straw	Cetyltrimethylammonium bromide	57.5 mg g^−1^	500 U g^−1^	45.1% (six cycles)	Wang et al. (2021a)
*T. hirsuta*	Polyvinylidene fluoride membrane	MWCNTs	30.4 mg cm^−2^	4.47 U cm^−2^	20% (five cycles)	Masjoudi et al. (2021)
*T. versicolor*	Wheat straw	No	-	-	-	Wang et al. (2021b)
-	Waste newspaper derived cellulose nanocrystals	No	64.94%	1.108 U mg^−1^	67% (six cycles)	Xing et al. (2021)
*Aspergillus sp.*	Microporous starch	No	-	-	-	Chen et al. (2021)
*A. oryzae*	MWCNTs	No	-	522 U g^−1^	-	Tavares et al. (2015)
*Bacillus subtilis*	Luffa sponge	Fe_3_O_4_ (Magnetic)	80 mg g^−1^	6.85 U mg^−1^	84.25% (10 cycles)	Zhang et al. (2020a)
*A. oryzae*	MWCNTs	Hydrothermal oxidation with HNO_3_	96%	20.5%	65% (five cycles)	Costa et al. (2019)
*T. versicolor*	Hollow mesoporous carbon nanospheres	NH_2_ (amino functionalize)	835 mg g^−1^	88%	60% (eight cycles)	Shao et al. (2019)
*T. versicolor*	MWCNTs	No	300 µg mg^−1^	0.2 U mg^−1^	-	Park et al. (2012)
*T. versicolor*	MWCNTs	HNO_3_	420 µg mg^−1^	0.3 U mg^−1^	-	Park et al. (2012)
*T. versicolor*	Graphene oxide	No	450 µg mg^−1^	0.7 U mg^−1^	-	Park et al. (2012)
*T. versicolor*	Pecan nutshells	FeCl_3_		-	-	Ramírez-Montoya et al. (2015)
*T. versicolor*	Pistachio shell	CaHPO_4_		-	-	Ramírez-Montoya et al. (2015)
*T. versicolor*	Pine nutshell	CaCl_2_		-	-	Ramírez-Montoya et al. (2015)
*T. versicolor*	Mesoporous carbon capsules	Fe_3_O_4_ (Magnetic)	-	-	-	Valle-Vigón and Fuertes, (2011)
*A. oryzae*	MWCNTs	HNO_3_	98%	250 U mg^−1^	-	Silva et al. (2014)
*A. oryzae*	MWCNTs	No	75%	600 U mg^−1^	-	Silva et al. (2014)
*B. subtilis*	*Prosopis juliflora* bark	H_3_PO_4_	-	-	40% (eight cycles)	Thiyagarajan et al. (2020)
*Myceliophthora thermophila*	MWCNTs	Cellulose nitrate	0.286 U mg^−1^		95% (10 cycles)	Othman et al. (2016)
-	Graphene oxide	Zeolite	350 mg g^−1^	-	95% (five cycles)	Thiyagarajan et al. (2020)
*T. versicolor*	Polyvinyl alcohol/chitosan	MWCNTs	907 mg g^−1^		80% (seven cycles)	Xu et al. (2015)
*T. versicolor*	CNTs	No	-	-	-	Zhang et al. (2020b)
*T. versicolor*	Electrospun fibrous membranes	MWCNTs	-	4.53 U mg^−1^	-	Dai et al. (2016)
*T. versicolor*	Pinewood	H_2_SO_4_/HNO_3_	26%	1.84 U mg^−1^	11% (seven cycles)	Naghdi et al. (2017)
*A. oryzae*	Granular activated carbon (GAC)	HCl	10 mg g^−1^	33 µM_DMP_ min^−1^ ^a^	-	Nguyen et al. (2016)
*T. versicolor*	CNTs	Polymethacrylate	-	-	90% (10 cycles)	Lai et al. (2019)
*T. pubescens*	Graphene Platelet	Polymer hydrogel	-	-	-	Ormategui et al. (2015)
*T. versicolor*	Pinewood	H_2_SO_4_/HNO_3_	-	4.95 U g^−1^	10% (seven cycles)	Naghdi et al. (2018)
*A. oryzae*	Graphene sheet	H_2_SO_4_/ethanol	179.12 mg g^−1^	-	-	Skoronski et al. (2017)
-	Activated carbon- Polyvinyl formal	H_2_SO_4_	-	-	51% (seven cycles)	Ma et al. (2017)
-	SWCNTs		0.8 mg g^−1^ for Lac	8 U mg^−1^ for Lac		Li et al. (2013b)
			0.9 mg g^−1^ for HRP	110 U mg^−1^ for HRP		
-	Pinewood	Citric acid	14.58 U g^−1^	10 U ml^−1^	-	Lonappan et al. (2018a)
-	Almond shell	Citric acid	24.3 U g^−1^	10 U ml^−1^	-	Lonappan et al. (2018b)
-	Pig manure	Citric acid	31.4 U g^−1^	-	-	Lonappan et al. (2018a)
-	Pinewood	Citric acid/Glutaraldehyde	20 U g^−1^	-	43% (five cycles)	Lonappan et al. (2018b)
-	Almond shell	Citric acid/Glutaraldehyde	30 U g^−1^	-	41% (five cycles)	Lonappan et al. (2018a)
-	Pig manure	Citric acid/Glutaraldehyde	40 U g^−1^	10 U ml^−1^	40% (five cycles)	Lonappan et al. (2018b)
*T. versicolor*	Graphene oxide	CuFe_2_O_4_	14.16 mg g^−1^		80% (10 cycles)	Rouhani et al. (2018)
	Graphene oxide	Fe_3_O_4_	-	-	60% (10 cycles)	Chen et al. (2017)
*T. versicolor*	C_60_ powder	No	1.2 mg g^−1^	10% of initial activity	-	Pang et al. (2015)
*T. versicolor*	MWCNTs	No	1.3 mg g^−1^	40% of initial activity	-	Pang et al. (2015)
*T. versicolor*	Oxidized MWCNTs	No	1.4 mg g^−1^	38% of initial activity	-	Pang et al. (2015)
*T. versicolor*	Graphene oxide	No	1.3 mg g^−1^	65% of initial activity	-	Pang et al. (2015)
*Aspergillus sp.*	Graphene oxide nano-sheets	No	150 mg g^−1^	-	-	Kashefi et al. (2019)
*T. versicolor*	Graphene oxide	Polyethersulfone	1 mg g^−1^	0.108 U mg^−1^	-	Xu et al. (2018)
*T. versicolor*	Graphene oxide	N_α_,N_α_-Bis(carboxymethyl)-l-lysine hydrate (NTA-NH_2_)	177 mg g^−1^	-	(89.4% (10 cycles)	Samak et al. (2018)
*T. pubescens*	Reduced graphene oxide	Xerogels	-	20 U ml^−1^	-	Rodriguez-Couto et al. (2014)
*T. pubescens*	Reduced graphene oxide	Hydrogel	-	4.33 U ml^−1^	-	Rodriguez-Couto et al. (2014)
*A. niger*	Graphene oxide	alginate	-	85 U g^−1^	-	Sharifi-Bonab et al. (2016)
*T. versicolor*	Graphene oxide	Fe_3_O_4_	-	86% of initial activity	-	Rouhani et al. (2021)

a Oxidation with 2,6-dimethoxy phenol (DMP).

#### Biochar as an Inducer of Laccase Production

In a biochar-based medium for laccase production, laccase can adsorb onto biochar or some of its components can be released in the culture medium and absorbed by the fungus. In both cases, as discussed in other sections, these organic and inorganic components in the biochar exert regulatory actions on laccase production, either as promoting or inhibiting agents (Giller et al., 1998). Due to its physicochemical characteristics, *i.e*., its high porosity and hydrophobicity (Taskin et al., 2019b), biochar can demonstrate high affinity for organic and inorganic contaminants (Taskin et al., 2019b; Fernandez-Sanroman et al., 2020). This property allows its use as a sorbent of organic or inorganic pollutants for soil amendments (Taskin et al., 2019b). Biochar has also been used in wastewater as additive/support media during anaerobic digestion, filtration matrix for the removal of suspended matter, heavy metals, or pathogens (Madadi and Bester, 2021).

The presence in biochar of bioavailable organic components like hydrophilic compounds and thermally labile fractions (Rombolà et al., 2016), adsorbed volatile organic compounds (Spokas et al., 2011), and polycyclic aromatic hydrocarbons (Buss et al., 2015) is well established. Many inorganic compounds including essential elements for the improvement of fungal laccase production such as Cu, Mn, or Fe have also been found in the biochar structure (see below). On the other hand, some of these compounds are potentially toxic and can be detrimental to laccase production or immobilization (Singh et al., 2010; Zhang G. et al., 2018). In some cases, it all depends on biochar level in culture media (Taskin et al., 2019b). Ultimately, the use of biochar as a substrate for laccase production or immobilization remains an open question.

Regarding carbon-based stimulation of WRF enzyme production, Liu et al. (2019) investigated the impact of single-walled carbon nanotubes, graphene and oxidized graphene (graphene oxide, GO) on the extracellular LME activities of a *Cladosporium sp*. strain, using a SmF with basal medium made of peptone and yeast extracts. It was found that, among the three carbon-based materials tested, single-walled carbon nanotubes and graphene increased laccase production, while GO caused a slight decrease in laccase activity (Liu et al., 2019). The effects on laccase expression of two carbon-based materials, *i.e.*, biochar (BC) and hydrochar (HC) prepared from four feedstocks were also studied using *T. versicolor, P. ostreatus* and *P. eryngii* strains (Taskin et al., 2019a). At two different doses (0.4 and 2% w/v), the two materials significantly stimulated laccase production and increased its activity for *T. versicolor* and *P. eryngii* strains, but *P. ostreatus* did not release any detectable laccase. Hence, BC from red spruce pellets at 0.4% w/v and HC from urban pruning residues at 2% w/v have promoted *T. versicolor* laccase activity by 6.4 and 21-fold with respect to the controls, respectively. Similarly, BC from vine pruning residues at 0.4% w/v and HC from urban pruning residues at 2% w/v induced a 6.4- and 21-fold increase in *P. eryngii* laccase activity over controls, respectively. Despite the promoting impacts of BC on laccase production, some inhibitory effects were noticed in connection with higher doses of BC (2%, w/v) in laccase expression by *T. versicolor* and *P. ostreatus* (Taskin et al., 2019a). On the other hand, Ascough et al. (2010) previously found depressive effects of BC at concentrations as low as 0.5% (w/v) on the growth of *P. pulmonarius* and *T. versicolor*. As for the effects of microelements such as Cu, Fe and Mn, Taskin et al. (2019a) could relate laccase expression induction to high levels of Fe (about 4.3 mM) and Mn (2.5 mM) in BC. In contrast, the absence of Mn, coupled with the presence of As, Pb, and Cl at relatively high levels, may have contributed to the decrease of laccase expression by *P. ostreatus* at both BC doses.

Lonappan et al. (2018a) immobilized laccase on BC from three different feedstocks, *i.e.,* pine wood (BC-PW), pig manure (BC-PM) and almond shell (BC-AS) produced in different pyrolysis conditions, for diclofenac elimination. The specific surface areas of the three BCs, determined using the Brunauer, Emmett, and Teller (BET) method were 14.1 m^2^ g^−1^ (BC-PW), 46.1 m^2^ g^−1^ (BC-PM) and 17 m^2^ g^−1^ (BC-AS), respectively. The BCs exhibited different surface texture, morphology, surface chemistry and functional groups. In addition, they demonstrated good results in covalent laccase immobilization, with BC-PM being the best immobilization support, mostly due to its higher specific area. In a similar study, two BCs prepared from maple (MB) and spruce (SB) were used as supports for laccase immobilization and for chlorinated biphenyl removal in wastewater (Li et al., 2018). FT-IR, SEM and BET analyses showed a honeycomb structure in the MB with a specific area of 613.6 m^2^g^−1^ and pore volume 0.695 cm^3^g^−1^ while SB exhibited 86.3 m^2^g^−1^ specific area and 0.065 cm^3^g^−1^ pore volume. Maple-based BC displayed the higher immobilization yield (Li et al., 2018).

As mentioned earlier, several studies have demonstrated the potential of ethanol to induce laccase production (Meza et al., 2005; Manavalan et al., 2013; Valle et al., 2014, 2015). Furthermore, due to its antimicrobial activity, ethanol has also been used as inactivating agent of competing fungal strains (Peters et al., 2013; Lucas et al., 2017) and other undesired microorganisms. In addition, ethanol is a safe, stable, and affordable solvent that can easily permeate the BC structure. Therefore, ethanol-based sterilization of BC and the subsequent use of the soaked BC as a substrate and carrier for laccase production and immobilization may be considered as an attractive means of enhancing the expression of specific fungal laccases. More generally, BC could be soaked in inducer solutions (e.g., copper containing solution) to serve as a complete culture medium of laccase production.

### Carbonaceous Materials as a Support for Laccase Immobilization

Carbon-based materials have been identified as effective and valuable supports in enzyme immobilization and have been implemented especially in the past two decades (Daoud et al., 2010). Carbon-based materials usually have fully developed pore structures with adequate pore size and high surface area (up to 1000 m^2^ g^−1^) which make them appropriate candidates for enzyme immobilization (Zdarta et al., 2018a). Besides these properties, carbon-based materials contain a great number of functional groups (*i.e.* carboxyl, and hydroxyl) on their surface which makes them ideal candidates for covalent and adsorption immobilization (Zdarta et al., 2018b).

### Graphene and Graphene-Related Materials

Graphene-based materials are promising immobilization supports due to inherent properties such as their high surface area (approximately 2630 m^2^ g^−1^), and functional groups such as epoxide, carboxylic, and hydroxyl on their surface (Daneshmandi et al., 2021; Karthik et al., 2021). Graphene materials have been used for enzyme immobilization through adsorption or covalent methodologies (Zhou et al., 2021). For instance, Skoronski et al. (2017) studied immobilization of laccase from *Aspergillus sp.* on commercial graphene nanoplatelets as a support (Skoronski et al., 2017). In this study, laccase activity immobilized on graphene through adsorption and covalent binding was evaluated. For covalent binding, graphene was modified through a nitration process to ensure that -NH_2_ groups would be created on its surface. Then using glutaraldehyde as a cross-linker agent, laccase was immobilized on the modified graphene surface. The obtained results demonstrated that laccase immobilized on graphene covalently could maintain its activity (around 80% of initial activity) after six cycles while the other forms of immobilizations such as adsorptive immobilization could not keep the activity after five cycles of operation.

Two other forms of graphene are graphene oxide (GO) and reduced graphene oxide (rGO). GO could be prepared through various methods such as Brodie, Staudenmaier, and Hummers processes in which graphite layers are separated followed by an oxidation step with strong oxidizing agents (Adeel et al., 2018). The oxidation step increases the distance between layers (Adeel et al., 2018). In a study on GO, atomic force microscopy (AFM) analysis demonstrated that a fully enriched surface of GO with abundant oxygen-containing functional groups such as epoxide, hydroxyl, and carboxyl could possibly enable laccase to attach to GO sheets without the need for further modification or cross-linking reagents (Zhang J. et al., 2010). In addition, it was demonstrated that as the extend of reduction of GO increases, the obtained support would have better enzyme loading capability and stability (Skoronski et al., 2017; Catania et al., 2021; Olabi et al., 2021). Kashefi et al. (2019) investigated laccase immobilization on GO covalently. Through addition of glutaraldehyde, it was demonstrated that in the final biocatalyst laccase obtained from *Aspergillus sp.* was covalently attached to GO sheets. Additionally, the final catalyst maintained 75% of laccase initial activity after six cycles.

Reduced GO is produced through removing oxygen functional groups from GO using different methodologies such as thermal reduction (Mcallister et al., 2007), photo-reduction (Zhang Y. et al., 2010), electrochemical reduction (Ramesha and Sampath, 2009), microwave reduction (Zhu et al., 2010), and chemical reduction (Stankovich et al., 2007; Olabi et al., 2021). Various reducing agents can be implemented in each procedure such as hydroiodic acid, ascorbic acid, hydrazine, and NaBH_4_ (Pei and Cheng, 2012; Lavin-Lopez et al., 2017). In a study by Patel et al. (2017) laccase was immobilized on a composite support produced through doping Fe_3_O_4_ on the rGO surface. The results illustrated that laccase stability was improved 15-fold at room temperature. Furthermore, the biocatalyst maintained 92% of initial activity after 10 cycles (Patel et al., 2017). Table 5 describes each type of graphene and its properties.

**TABLE 5:** Advantages and disadvantage of graphene materials (Catania et al., 2021).

	Advantage	Disadvantage
Graphene	Good control of functionalization	High production cost
		Small-scale production
GO	Water dispersibility	Poor control of functionalization after preparation
	Polar functionalization	
	Cheap	
	Easy to use	
rGO	Lower price compared to graphene	High production cost
	Good control of functionalization	

#### Carbon Nanotubes

Carbon nanotubes (CNTs) or buckytubes are hollow cylinders in which carbon atoms are located in hexagonal arrangements (Assi et al., 2021). Since CNT materials are formed from graphene sheets, they demonstrate similar properties to graphene materials like thermal and chemical stability, high tensile strength, and biocompatibility (Karthik et al., 2021). However, graphene atoms are in a two-dimensional arrangement while carbon atoms of CNTs are in a one-dimensional arrangement (Anzar et al., 2020). Moreover, CNTs exhibit radical breathing mode (RBM) in Raman spectrum which is unique to CNTs in comparison with other carbon systems, where all of the carbon atoms move in the radial direction synchronously thus generating an effect similar to breathing (Lei et al., 2011). CNTs can be formed through three different methods, i.e., arc discharge method, laser ablation method and chemical vapor deposition procedure. Commonly, two forms of CNTs can be developed: single wall carbon nanotubes (SWCNTs), and multiple wall carbon nanotube (MWCNTs).

- Single-walled carbon nanotubes:

SWCNTs may be developed from a single graphene sheet rolling upon itself (1–2 nm diameter) (Sabzehmeidani et al., 2021). SWCNTs were first reported in 1993 (Karthik et al., 2021). They have unique properties such as strong covalent bonding, one-dimensional structure, and nanometer size (Liu et al., 2015). Based on how graphene sheets are rolled up, two forms of SWCNTs can be obtained: a zigzag structure, and an armchair structure (Shoukat and Khan, 2021).

- Multi-walled carbon nanotubes:

MWCNTs are prepared by rolling up multiple layers of graphene sheets on themselves (Ali et al., 2021). Based on the number of graphene tubes being rolled up, MWCNT diameter varies from 2 to 50 nm (Ibrahim, 2013). The simplest form of MWCNT is a double-walled carbon nanotube (DWCNT) (Karthik et al., 2021).

Recently, studies on enzyme immobilization over CNTs have increased rapidly since these materials have high surface area, capability of enhanced enzyme loading, and low mass transfer hindrances. For instance, in a study conducted by Xu et al. (2015) laccase was immobilized on a novel composite membrane (polyvinyl alcohol/chitosan/MWCNTs) (Xu et al., 2015). The immobilization was completed through surface modification of the membrane with glutaraldehyde. The final product was shown to maintain 80% of initial laccase activity after seven cycles of operation. As mentioned previously, industrial application of nanoparticles due to their small sizes could be challenging, especially their handling in the environmental arena. Most studies in the field of enzyme immobilization on graphene and carbon nanotubes are related to biosensor applications. In addition, plasma based treatment/production of CNTs may result in better immobilization/loading of laccase. Plasma based treatments are non-polluting in nature and can provide a wide range of functional groups (Ruelle et al., 2011). To the best of our knowledge, this technique has not been used for the immobilization of laccase on plasma treated CNTs. However, Othman et al. (2016) used MWCNTs synthesized using plasma enhanced chemical vapor deposition for the immobilization of laccase (Othman et al., 2016)

#### Activated Carbon

Activated carbon (AC) denotes amorphous carbonaceous materials with good chemical and physical characteristics (Barroso Bogeat, 2021). Its high surface area (600–1300 m^2^ g^−1^) with large number of contact sites makes activated carbon a valuable support for enzyme immobilization (Karthik et al., 2021). Previous studies have demonstrated that natural activated carbon or functionalized activated carbon with HCl could act as a support in laccase immobilization (Sirisha et al., 2016). Recently mesoporous activated carbon with large contact sites has been using for laccase immobilization as well as acid protease and acid lipases immobilization (Ganesh Kumar et al., 2010; Datta et al., 2013). In a study, activated carbon fibers modified with dopamine was utilized as a support for laccase obtained from *Aspergillus sp.* immobilization (Zhang C. et al., 2018). The results indicated that the biocatalyst had the capability of maintaining its activity (around 60% of initial laccase activity) after six cycles of operation while free laccase only kept 40% of initial activity after the same number of operations (Zhang C. et al., 2018). Table 4 presented various studies of immobilization of enzymes on carbon based materials.

#### Kinetic Parameters of Immobilized Laccase

Kinetic parameters such as Km, Vmax and the catalytic efficiency kcat/Km determine the catalytic action of enzymes. These parameters can vary considerably depending on the types of enzymes, support materials and process conditions. The Michaelis constant (Km) expresses the affinity of the laccase to the substrate. Vmax is the maximum reaction rate. Low apparent Vmax can result from mass transfer limitations and reduction in enzyme–substrate affinity after immobilization (Gahlout et al., 2017). The Vmax/Km ratio reflects the catalytic efficiency of the enzyme-substrate system. Some values of kinetic parameters related to free laccase and its immobilized counterparts formed using different techniques and carriers are reported in Table 6.

**TABLE 6:** Kinetic parameters related to different immobilization techniques and carriers used.

Laccase strain	Immobilization technique/carrier	Substrate specificity	Vmax μM/min	Km (mM)	Kcat (μmol s^−1^ g^−1^)	kcat/Km (L s^−1^ g^−1^)	References
Genetically modified *Aspergillus sp.*	Covalent bond/graphene oxide nanosheets	ABTS	45.88 ± 4.3	1.16 ± 0.07	82.36 ± 6.7	0.07 ± 0.005	Kashefi et al. (2019)
	Free enzyme		62.11 ± 3.8	0.71 ± 0.06	103.52 ± 4.4	0.14 ± 0.01	
Non specified	Covalent immobilization on Zeolite nanoparticles	Direct Red 23	3270 ± 103	70.308 ± 4.29^a^			Mahmoodi and Saffar-Dastgerdi, (2020)
	Covalent immobilization on Graphite oxide-zeolite nanocomposites		7580 ± 130	118.702 ± 34.30^a^			
*Coprinus comatus*	Adsorption on Maple biochar	ABTS		2.68			Li et al. (2018)
	Free enzyme			0.223			
*T. versicolor*	Covalent immobilization on biochar	Catechol	38 ± 2	0.077 ± 0.012	0.045 ± 0.002	0.058 ± 0.001	Zhang and Hay, (2020)
	Recombinant *E. coli* strain expressing *B. subtilis*		44 ± 3	0.096 ± 0.013	0.057 ± 0.003	0.059 ± 0.005	
	Free laccase		43 ± 3	0.072 ± 0.011	0.053 ± 0.003	7.4 × 10^–2^ ± 6.0 × 10^–5^	
*Ganoderma cupreum*	Covalent immobilization on silica	ABTS		358	0.5		Gahlout et al. (2017)
	Free laccase			1234	0.19		
*T. versicolor*	Covalent immobilization on graphene oxide/CuFe_2_O_4_ nanocomposite	ABTS	26	1.8			Rouhani et al. (2018)
	Free laccase		56	1.3			
*B. subtilis*	Adsorption on magnetic carbon nanocarriers		9.72	0.09			Zhang et al. (2020a)
	Free laccase		8.51	0.11			
*T. versicolor*	Covalent immobilization on silica-chitosan support	ABTS	0.0034	0.008			Girelli et al. (2020)
	Free laccase			0.041			
*M. thermophila*	Covalent immobilization on poly (glycidyl methacrylate) microspheres	ABTS	395.1 ± 25.6	7.3 ± 1.2	658.51	90.21	Vera et al. (2020)
*T. versicolor*			110.2 ± 5.3	2.5 ± 0.5	146.95	58.15	
*Aspergillus* sp.			165.1 ± 9.2	5.4 ± 0.8	302.64	55.59	
*T. versicolor*	Covalent immobilization of laccase Fe_3_O_4_@SiO_2_@Kit-6 magnetite nanoparticles	ABTS	39.59 μmol/g/min	345.37			Amin et al. (2018)
	Free laccase		121.25 μmol/g/min	211.13			
*T. versicolor*	Covalent immobilization on magnetic silica microbeads	ABTS		64.3 ± 6.7	134.6 ± 6.7	2.10 ± 0.11	Arca-Ramos et al. (2016)
	Free laccase			38.5 ± 3.1	153.7 ± 1.3	4.00 ± 0.29	
*B. subtilis*	copper-Trimesic acid framework			89.398	0.159	562.251	Zhang et al. (2020b)
	Free Laccase			5.417	0.108	50.157	

a Mol.

#### Biodegradation of Organic Contaminants by Immobilized Laccase

A number of studies have been performed using immobilized laccases for the biotransformation of organic contaminants. Most of these studies have been conducted using synthetic wastewater, however a few of them also involved real wastewater, at laboratory or pilot scale. Due to the immobilized enzymes’ overall stability over free enzymes and their recyclability, they generally exhibited higher removal. Table 7 summarizes some of the very recent studies on the application of immobilized laccase for emerging contaminant removal.

**TABLE 7:** Removal of trace organic contaminants by immobilized laccase.

Laccase strain	Immobilization technique/carrier	Treatment media	Removal efficiency	References
*Aspergillus sp.*	Covalent immobilization on peanut shell	Isoproturon, Atrazine, Prometryn, Mefenacet, Penoxsulam, Nitenpyram, Prochloraz, Pyrazosulfuron-Ethyl and bensulfuron-methyl, in mixed solution	>54.5% in water in presence of syringaldehyde	Chen et al. (2019)
			20.9–92.9% in soil	
	Covalent immobilization in wheat straw		>65.9% in water in presence of syringaldehyde	
			14.7–92.0% in soil	
Genetically modified *A. oryzae*	Enzyme coupled with granular activated carbon (GAC)	Carbamazepine	52% carbamazepine	Nguyen et al. (2014)
		Diclofenac	63% diclofenac	
		Sulfamethoxazole	58% sulfamethoxazole	
		Atrazine	75% atrazine	
	Free enzyme		10% carbamazepine	
			21% diclofenac	
			9% sulfamethoxazole	
			14% atrazine	
*M. thermophila* and *P. eryngii*	Covalent immobilization on Stevensite and biochar	Synthetic wastewater containing oxytetracycline tetracycline chlortetracycline	100% removal in presence of ABTS as mediator	García-Morales et al. (2018)
		Synthetic wastewater containing sulfathiazole sulfadiazine	100% sulfathiazole removal	
			54% sulfadiazine removal in presence of ABTS	
*T. versicolor*	Covalent immobilization on biochar	2–4 dichlorophenol contaminated soil	64.6% removal	Wang et al. (2021a)
	free enzyme		44.4% removal	
*B. subtilis*	Adsorption on magnetic carbon nanocarriers	Synthetic wastewater containing Bisphenol A	100% removal	Zhang et al. (2020a)
	Free enzyme		62.70% removal	
*M. thermophila*	Covalent immobilization on functionalized multiwalled carbon nanotubes	Reactive Black 5 (RB5) decolorization	84.26% decolorization in presence of 1-hydroxybenzotriazole as mediator	Othman et al. (2016)
*T. versicolor*	Covalent immobilization onto micro-biochar	Diclofenac in wastewater	100% removal	Lonappan et al. (2018b)
*T. versicolor*	physical absorption (HMCs-Lac) and covalent binding on hollow mesoporous carbon spheres (HMCs) and amino-functionalized	Synthetic wastewater containing TCH and CPH	93.8, 97.6, and 99.1% TCH removal for HMCs-Lac, HMCs-NH2-Lac and HMCs-NH2-GTA-Lac in presence of syringaldehyde	Shao et al. (2019)
	HMCs-NH_2_-Lac and HMCs-NH_2_-GTA-Lac		98.1, 99.4, and 99.2% THC removal for HMCs-Lac, HMCs-NH2-Lac and HMCs-NH2-GTA-Lac in presence of 1-hydroxybenzotriazole	
*T. versicolor*	immobilization on to acrylate microbeads	Synthetic wastewater containing Methylene Blue dye (MB) and Carbaryl pesticide (CP)	100% removal of MB and CP in presence of acetosyringone as mediator	Bayramoglu and Arica, (2019)
*Pycnoporus sanguineus*	Covalent immobilization on titania nanoparticles functionalized with APTES	Acetaminophen (ACE) and diclofenac (DCF)	68% DCF after 8 h	García-Morales et al. (2018)
			90% ACE after 2 h	
*T. hirsuta*	Entrappment in alginate beads	Carbamazepine and acetaminophen in binary solution	40% CBZ	Hachi et al. (2017)
			70% ACE	

## Biochar as an Emerging “Carbon Negative” Carbonaceous Solid Support for Immobilization of Laccase

### Biochar Properties and Sustainability

BC is a porous carbonaceous solid residue that can be obtained through biomass conversion *via* hydrothermal and thermochemical processes such as pyrolysis and gasification in the absence of oxygen under various temperatures (Kuzyakov et al., 2009; Liu et al., 2019; Cheng et al., 2021; Madadi and Bester, 2021). BC production is adding value to the economy because in this process wastes and biomass residues can be recycled and reused as secondary resources (Janu et al., 2021). Moreover, BC is carbon negative (Glaser et al., 2009) and its production and application feeds directly into the circular and sustainable economy (Bolognesi et al., 2021). In comparison to activated carbon, BC can be obtained from various types of resources requiring less production energy (Madadi and Bester, 2021). Also, in contrast to activated carbon, BC production is a chemical-free process (Frišták et al., 2018; Madadi and Bester, 2021). The existence of large numbers of polyaromatic carbon groups on BC surfaces with abundant functional groups (carboxyl and hydroxyl) makes it an efficient and low-cost support for immobilization (Komkiene and Baltrenaite, 2016; Kong et al., 2017; Tong et al., 2019). Surface area, existence of functional groups with affinity to laccase and pore size are the crucial parameters affecting laccase immobilization on BC (Madadi and Bester, 2021). BCs with high surface area, activated sites, and the proper porous structure can be considered as a cost-effective candidate compared to activated carbons for enzyme immobilization (Madadi and Bester, 2021). The physical and chemical properties of BC are highly dependent on the feedstock and conditions of production (Barroso Bogeat, 2021; Madadi and Bester, 2021).

#### Feedstock Composition

BC sources can be divided into two categories, i.e., BCs produced from lignocellulosic materials and BCs produced from non-lignocellulosic materials (Stella Mary et al., 2016; Karim et al., 2019). Lignocellulosic biochars can be divided into three different subcategories namely: wood (hardwood or softwood), crop waste, and grass and leaves (Ippolito et al., 2020). Non-lignocellulosic biochars mainly come from sewage sludge, manure, and algae (Ippolito et al., 2020; Pandey et al., 2020). From lignocellulosic sources, corn, wheat straw, and rice/husk straw are commonly used (Ippolito et al., 2020). Regarding non-lignocellulosic sources poultry, pig, and cattle manure are the most common sources for biochar production (Ippolito et al., 2020). Feedstock significantly affect the carbon content, surface area, and functional groups of final products (Novak et al., 2019). Normally carbon content is proportionally related to biomass lignin content. Biochars produced from wood feedstock demonstrates higher carbon content compared to other sources (Wang et al., 2016). Biochars produced from manure normally have higher content of N, S, and P (Ippolito et al., 2020). In the terms of surface area, lignocellulosic biochars have higher surface and among different sources, wood-based biochar represent higher surface area (Lehmann and Joseph, 2009; Weber and Quicker, 2018). Biochar produced from manure usually have low surface area due to structural cracking or micropore blockage (Ahmad et al., 2014; Ippolito et al., 2017). Regarding functional groups, normally lignocellulosic biochars exhibit content of hydroxyl and carboxyl bonds on their surface (Pandey et al., 2020). However, manure-based biochars demonstrate amine groups on their structures (Leng et al., 2019). The amine content on biochars obtained from different biomasses is followed the pattern in order of wood biochars<crop biochars<grass biochars<manure biochars (Ippolito et al., 2020).

#### Pyrolysis Type

There are two kinds of pyrolysis, slow and fast. During slow pyrolysis, low temperature heating rate (0.01^–2^ Cs^−1^) would be implemented (Sohi et al., 2009). However, temperature heating rate would be higher than 2°Cs^−1^ in fast pyrolysis. Pyrolysis type would affect surface area and average particle size (Ippolito et al., 2020). Biochar produced through fast pyrolysis usually have higher surface area compared to biochars produced with slow pyrolysis; however, fast pyrolysis biochars demonstrate lower average particle size compared to slow pyrolysis biochars (Asadullah et al., 2010; Qambrani et al., 2017).

#### Pyrolysis Temperature

Temperature is considered as a significant parameter that affects biochar physiochemical properties. Biochar porosity and surface area would crucially change by pyrolysis temperature variation. Generally, at higher temperature, larger pore volume and surface area would be expected (Mendonça et al., 2017; Weber and Quicker, 2018). Pyrolysis temperature could also affect the content of functional groups and aromatic structure of biochar. Biochar produced at temperature above 500°C demonstrate lower amount of O- and H-containing functional groups (Janu et al., 2021). However, biochars produced below 500°C exhibits higher O- containing functional groups (Janu et al., 2021). For instance, Li X. et al. (2013) studied how variation in pyrolysis temperature could affect biochar properties. The obtained results from two-dimensional (2D) ^13^C nuclear magnetic resonance (NMR) demonstrated the lower aromaticity ratio (H/C) and lower polarity (O/C and (O+N)/C ratios. This could happen because at higher temperature, the carbon content would increase while H, N, and O contents would decrease (Li X. et al., 2013).

### Biochar Engineering

BC engineering is identified as a procedure to manipulate BC properties to enhance its surface area, porosity and the content of functional groups. BC could be engineered through physical and chemical modification procedures.

#### Physical Activation

In the physical activation approach, no chemical agents are implemented, and this methodology is considered as an economical and simple approach (Rajapaksha et al., 2016). Physical activation of biochar involves the use of gases such steam, CO_2_, and ozone at temperatures above 700°C (Jimenez-Cordero et al., 2015; Shen et al., 2015; Shim et al., 2015). This modification can be summarized into two steps: first biochar surface area is increased through modification of its unstructured parts and second its crystalline-C formation is improved (Jung and Kim, 2014, 2014; Cha et al., 2016). Park et al. (2016) studied the effect of steam modification on BC surface. In this study BC was produced from *P. tenera* at 500°C and steam modification was carried out at 700°C for 1 h. The results confirmed that while the surface area of untreated BC was close to zero, that of treated BC increased to 22 m^2^ g^−1^ (Park et al., 2016).

#### Chemical Activation

During chemical modification, BC is mixed with a chemical agent and through dehydration and oxidation, its properties can change (Xiang et al., 2020). Despite its drawback such as the high cost of chemicals, and inability to recover and reuse such chemical agents, this method has a higher efficiency compared to physical activation (Cha et al., 2016). Chemical treatment of BC is achieved using strong acids such as H_3_PO_4_, HCl, and H_2_SO_4,_ and strong bases such as KOH, NaOH, and NH_3_ (Cha et al., 2016; Zhang C. et al., 2020; Pandey et al., 2020).

Acid treatments normally promote the emergence of oxygen-containing functional groups together with increasing surface area (Rajapaksha et al., 2016). In a study of covalent laccase immobilization on modified BC Lonappan et al. (2018b) used raw BC from pinewood, pig manure, and almond shell. Through BC modification with citric acid, more carboxylic groups were observed on its surface compared to untreated BC (Lonappan et al., 2018b).

BC alkalinization enhances non-polarity with increasing surface area and functional group content. Jin et al., 2014) studied the effects of KOH on the BC produced from municipal solid wastes (Jin et al., 2014). FTIR analysis demonstrated that the number of hydroxyl and carboxyl groups on the surface of treated BC was increased (Jin et al., 2014). In addition, surface area was increased from 14.4 m^2^ g^−1^ for raw BC to 49.1 m^2^ g^−1^ for treated BC (Jin et al., 2014).

### Specific Properties of Biochar for Immobilization of Enzymes

Previously, several studies were carried out on the “carbon negative” biochar to be used as a sustainable and green solid support for the immobilization of laccase (Kuzyakov et al., 2009). Porosity, existence of functional groups, stability, and surface area are important BC properties which could affect immobilization. Previous studies have been conducted to illustrate how feedstock, activation processes, and pyrolysis temperature could affect these properties. For instance, Jin et al. (2014) investigated the effect of chemical activation on BC produced from municipal solid wastes. The obtained results confirmed that KOH activation would increase the surface area from 14.4 m^2^ g^−1^ to 49 m^2^ g^−1^ (Jin et al., 2014). In another study, Kloss et al. (2012) studied how feedstock could affect surface area. The results illustrated that BCs derived from wood biomass often have higher surface area (Kloss et al., 2012). Furthermore, Zhao L. et al. (2017) studied the effect of pyrolysis temperature on physicochemical properties of produced BCs from apple tree branches. The final results explained that surface area increases with increasing pyrolysis temperature (Zhao S.-X. et al., 2017). More details on BC properties are given in the review by Madadi and Bester (2021).

As a waste management alternative, immobilization of enzymes on BC paves a sustainable pathway in environmental management. However, the disposal/management of used catalyst (i.e. enzyme immobilized on BC) is a potential concern. Despite the environmental friendliness and effectiveness of the BC-laccase catalyst, the disposal of the used catalyst must be carried out properly otherwise the used catalyst itself will end as another potential “emerging contaminant.” Consequently, the used catalyst could be valorized as a fertilizer in soil given the proved ability of BC in fertilizing agricultural lands (Ding et al., 2016). The unused enzyme present on the BC surface can further eliminate pesticides and other organic contaminants present in the soil. In addition, the accumulated nutrients on BC after its application in wastewater treatment (given that wastewater also contains several nutrients (Kaetzl et al., 2020)) will reach the soil as well. Therefore, re-using already utilized BC-enzyme catalyst after wastewater applications is bound to have a considerable positive effect as a soil amendment.

On the other hand, these BC-based biocatalysts could adsorb organic, inorganic and biological contaminants that could have negative impacts on the yield or the crop quality. Thus, the potential impacts as well as the fate of the adsorbed contaminants at the surface of BC should be further studied. Nonetheless, certain contaminants could be further degraded by the residual laccase present on the biochar surface.

## Conclusions, Current Research Challenges and Future Perspectives

This review provides a survey on the recent developments of laccase production, immobilization techniques, and application of carbon-based materials as supports.

Low productivity, low stability and limited reusability are the major concerns which challenge the industrial production and application of laccase. Although past studies have concentrated on enhanced laccase production through various methods and then through its immobilization, concerns regarding the cost-effectiveness of these approaches still exist and raise questions regarding their industrial feasibility.

• In the recent past, co-culture has been studied as an effective strategy for the enhanced production of laccase (Chan-Cupul et al., 2016). However, to be a successful process this approach requires the compatible coexistence of the different microbial species involved. Optimization of this process is often challenging. More studies are required to further elucidate the complex pathways behind the co-culture approach for laccase production.
• Inducers were previously proven to be a factor for the enhanced production of laccase if added at the correct concentration. Inducers such as Cu, 2,5-xylidine, guaiacol, etc. enhance laccase production and are usually added in the form of a more complex medium ingredient containing these elements/chemicals. However, addition of expensive chemicals may endanger process economics. Thus, waste/residual materials/inexpensive materials such as biochar can be an economically attractive alternative for industrial production. Nevertheless, it has to be noted that the process must be optimized on the basis of the inducer concentration in the residual materials. In addition, the choice of these “residual material-based” inducers must be made wisely as the presence of potential toxic molecules can inhibit fungal growth and thus laccase production.
• For a given species, the culture media composition is one of the key determining factors which dictates the overall productivity of the process. This is also the most cost-intensive factor (Alessandrello and Vullo, 2016). Thus, inexpensive “culture media alternatives” could significantly reduce the overall cost of the process. In the recent past, the quest for inexpensive and sustainable alternatives for growth media/substrates has resulted in various waste/residual materials such as olive tree saw dust, olive pomace, apple pomace, etc. However, fermentation using these materials must be carried out under solid state conditions for which process control such as maintaining pH, mixing and aeration cannot be easily obtained. On the other hand, for liquid residual materials, submerged fermentations can be carried out and thus adequate process control can be implemented. Research in this domain is minimal and further studies in these directions will improve the sustainability and cost-effectiveness of the overall process at industrial scale.
• Future studies should be carried out in the direction of concomitant enzyme production and immobilization. Instead of completing each procedure separately, the development of procedure using feedstocks which can satisfy both enzyme production enhancement and efficient immobilization would be cost-effective and efficient.

Free laccase is comparatively unstable and expensive and thus it has to be immobilized/cross linked for real life applications. Immobilization of laccase over solid supports could significantly enhance the capability of laccase to maintain its activity over time and its resilience to operational conditions (such as temperature, pH, and exposure to different chemical agents) (Shakerian et al., 2020). Various immobilization methods such as entrapment, adsorptive and covalent immobilization and cross linking have been employed and extensively studied in the past. For immobilization supports the particle size, specific surface area, porosity, mechanical properties and surface functional groups play important roles in the extent of immobilization. Various immobilization supports were studied in the past and application of carbonceous materials is interesting owing to their organic/renewable origin and nature. In particularly, activated carbon, carbon nano tubes, and graphene are well known immobilization supports and which have been used frequently for laccase immobilization.

The application of biochar as an immobilization support for laccase is under-explored and this review is an attempt to summarize the existing studies and further explore biochar’s potential as an immobilization support for laccase. Because of its carbon negative nature (Glaser et al., 2009) application of biochar can be a further step towards sustainability and integration into the circular economy. As previously described, particle size, specific surface area, porosity, mechanical properties and surface functional groups play important roles in the extent of the immobilization. For biochar these properties are often dictated by feedstock composition and method of production. Thus, properly designed and engineered biochar materials can result in excellent immobilization/loading of laccase on their surface. Moreover, biochar activation can be an effective tool for enhanced laccase loading/immobilization.

In summary, the following gaps in research, technological challenges and perspectives for future studies may be noted:

• The application of BC itself as a substrate for fungi can be interesting and challenging at the same time. The limiting factors here may reflect nutrient deficiencies and presence of growth inhibitors. However, further research in this direction could be profitable, given the sustainable and cost-effective nature of BC.
• The minimal cost of BC production and its special features such as the existence of functional groups, porosity, and surface area are key positive factors in considering BC as an immobilization support. However, raw BC is not sufficiently diversified in terms of functional groups. Although a number of past studies have focused on BC functionalization, future work should be directed towards increasing amino groups on the BC surface to enhance the potential of chemical immobilization of laccase and similar enzymes.
• While glutaraldehyde has been identified as a common and efficient cross-linker for chemical immobilization, it can be harmful for the environment or exposed workers. Therefore, future studies should be concentrated to identify green and environmental friendly alternatives.
• BC is rich with many molecules which can act as potential mediators for laccase-based elimination of ECs. Thus, BC-immobilized laccase has already the potential for enhanced elimination of ECs. In addition, further engineering BC with functional groups which can act as mediators for laccase-based elimination of ECs can be a promising area for further research.
